# Supplementary material for: Activation of Lineage Regulators and Transposable Elements across a Pluripotent Spectrum
Source: Stem Cell Reports. 2017 Jun 9;8(6):1645–58. doi: 10.1016/j.stemcr.2017.05.014 (PMC5470235; doi:10.1016/j.stemcr.2017.05.014)
Supplement: Document S1. Supplemental Experimental Procedures and Figures S1–S5 [file mmc1.pdf]

Stem Cell Reports, Volume *8*

Supplemental Information

Activation of Lineage Regulators and Transposable Elements across a Pluripotent Spectrum

Jamie A. Hackett, Toshihiro Kobayashi, Sabine Dietmann, and M. Azim Surani

EXTENDED EXPERIMENTAL PROCEDURES

Culture of ESCs

Undifferentiated mouse ESCs from 129 (129X1/SvJ) ('CES5'), B6 (C57BL6/J) ('BK2'), *Stella*-GFP/2C::tdTomato, *Blimp1*-GFP, ΔPE-*Oct4*-GFP and *Sox1*-GFP (transgenic derived) strains were maintained on gelatin-coated dishes in the specified culture mediums, for at least 5 passages (see Fig 1A). ESC were propagated in a humidified 37°C chamber supplemented with 5% CO_2. ESC culture media was replaced with fresh media every day and cells were routinely passaged every 2-3 days. For basal medium, N2B27 medium was prepared according to a published protocol or purchased as NDIF 227 from Takara bio (Ying et al., 2008). For GMEM or KO-DMEM based media, each medium purchased from Thermo scientific was supplemented with 0.1 mM 2-mercaptoethanol (Thermo scientific), 0.1 mM non-essential amino acids (Thermo scientific), 1 mM sodium pyruvate (Thermo scientific), 1% L-glutamine (Sigma) and penicillin/streptomycin (Thermo scientific). For signalling or supplementation, 10% fetal bovine serum (Thermo scientific), 10% KSR (Thermo scientific), 1 μM MEK inhibitor PD0325901 (MACS), 3 μM GSK3 inhibitor CHIR99021 (MACS), and 1,000 U/ml of mouse LIF (Cambridge Stem Cell Institute) are added respectively according to the condition.

Embryo Culture and Manipulation

Preparation of C57BL6/J x BCF1 intercrossed embryos was carried out according to standard protocols. In brief, 8-cell/morula stage embryos were collected in M2 medium (sigma) from the oviduct and uterus of mice 2.5 days *post coitum* (dpc). For micro-manipulation, sorted *Blimp1*-GFP ESCs were suspended in ESC culture medium. A piezo-driven micro-manipulator (Prime Tech, Tokyo, Japan) was used to drill zona pellucida under the microscope and 1 or 5 ESCs were introduced into the space between zona pellucida and blastomeres of 8-cell/morula stage embryos. After injection, embryos were cultured until the next day in KSOM medium (Millipore). Morula or early blastocysts were subsequently transferred into the uteri of pseudopregnant recipient MF1 female mice (2.5 dpc).

ESC Differentiation Protocols

Specification of primordial germ cell-like cells (PGCLC) was performed as previously described, with minor modifications (Hayashi et al., 2011). ESC were maintained in the indicated culture medium on gelatin coated plates. Induction into epiblast-like cells (EpiLC) was performed by passaging $1.2x10^5$ ESC onto fibronectin coated 12 well plates containing N2B27 supplemented with 12.5ng/ml FGF2, 20ng/ml Activin-A (both Cambridge Stem Cell Institute) and 1% KSR for 40 hours. PGCLCs were subsequently specified in embryoid bodies formed in ultra-low attachment 96-well plates (Corning) containing GK15 supplemented with 500ng/ml BMP4, 500ng/ml BMP8, 50ng/ml EGF, 100ng/ml SCF (all R&D Systems) and 1,000 U/ml mouse LIF. Induction of monolayer neuro-ectoderm differentiation was performed as previously described (Ying et al., 2003). Briefly, *Sox1*-GFP ESC maintained in the indicated ESC culture medium were washed with PBS thrice, and passaged to gelatin-coated 6-well plates containing NDIF 227 (Takara) without supplements at optimal density.

Analysis of *Blimp1*-GFP ESC

To analyse the percentage of *Blimp1*-GFP subpopulation by FACS, cells were trypsinized and suspended in PBS containing 3% FCS. To check CD31 expression, cells were stained with APC-conjugated anti-CD31 antibody (eBioscience) for 30-60 min on ice. Cells were analyzed by FACS LSRFrotessa (BD Bioscience) or sorted by Moflo (Beckman Coulter). All data were

re-analyzed by Flowjo software. For bulk colony formation assay, 10,000 cells were sorted and seeded on a gelatin coated well of 24 well plate. After 5 days, cells were fixed and stained with Alkaline Phosphatase Detection Kit (Sigma) according to manufacturer's protocol. For single cell colony formation assay, single Blimp1-GFP positive or negative cells were sorted into ESC medium on gelatin coated well of 96 well plate. After 5 days, numbers of well has undifferentiated ESC colony were counted. For signaling inhibition experiments, 10 uM Jak inhibitor I (sigma); 5 uM LY294002 (Sigma), were added to N2B27+2i/L medium and cells analyzed by FACS after 2-3 days culture. For screening TFs to suppress *Blimp1*-GFP positive population, a PiggyBac vector containing *CAG* promoter, genes shown in Fig 5B and *IRES-Puro* cassette were co-transfected with a PBase expression vector into *Blimp1*-GFP ESCs cultured in 2i using lipofectamine 2000 (Thermo scientific). Two days after transfection, 1.0ug/ml puromycin was added to medium to select cells that stably express the transgene. Cells were analysed 7-10 days after transfection.

Generation and Rescue of *Klf4* KO ESCs

For *Klf4* KO ESCs, the CRISPR/Cas9 double nickase system was used. Two guide RNAs coding (5'-GGCAGGGCCGCTGCTCGCCG-3' and 5'- CAGCTATCCGATCCGGGCCG-3') were inserted into *BsgI* restriction enzyme site of pX335 vector. These plasmids were co-tranfected with vector containing Puromycin resistance gene into *Blimp1*-GFP ESCs using lipofectamine 2000. Two days after transfection, 1.0ug/ml puromycin was added to the medium and drug resistant (transfected) cells were selected for a further 2 days. After the selection, ESC colonies were picked up and screened for homozygous KO by PCR, Western blot (data not shown), and IF. To rescue *Klf4* KO ESCs, a variant of Shield1/destabilized domain (DD) (Clontech) inducible expression system was used. A PiggyBac vector containing *CAG* promoter, DD fused HA tagged *Klf4* and *IRES-Puro* cassette were co-transfected with PBase expression vector into *Klf4* KO ESCs using lipofectamine 2000 (Thermo scientific). After puromycin selection, the resistant colonies were picked and the expression level of *Klf4* was validated by Western blot.

Generation of *Stella*-GFP & 2C::tdTomato double-reporter ESCs

For construction of the targeting vector for the *Rosa26* locus, a splice accepter sequence, Neomycin resistance gene with pA and insulator sequence, *2C-tdTomato* cassette amplified from 2C::tdTomato Reporter (a gift from Prof. Samuel Pfaff), and *IRES-Puro* were inserted into *XbaI* site of pROSA26-SwaI with the InFusion cloning kit (Takara Bio). For gene targeting, electroporation was carried out using *Stella*-EGFP transgenic (shorter SH6 transgene construct) ESCs. Briefly 4 x 10^6 ESC suspended in PBS were mixed with 20 μg linearized targeting vector, and then, were transferred to a Gene Pulser cuvette (Bio-Rad, Richmond, CA). Electroporation was carried out at 230 V, 250 μF in Gene Pulser equipment (Bio-Rad). After electroporation, ESCs were seeded on gelatin-coated dish, and 24 h later, 400μg/ml G418 (Sigma) was added to the culture medium. After drug selection, colonies were picked up and screened for correct targeting by PCR.

Immunofluorescence

Cultured ESCs were fixed in 4% paraformaldehyde for 10 min at room temperature. After permeabilisation with 0.25% triton-X/PBS for 10 min, ESC were blocked with 0.1% triton-X/ MAXblock™ blocking medium (Active Motif) for 30 min, and subsequently incubated with primary antibodies for 1-2 hr at RT or overnight in a cold room. Primary antibodies against

NANOG (eBioMLC-51; eBioscience), OTX2 (AB9566; abcam), PAX6 (PRB-278P; Covance), BLIMP1 (6D3; eBioscience), GATA4 (EPR4768; abcam), SOX17 (AF1924; R&D systems), KLF4 (AF3158; R&D systems), and EGFP (GF090R; Nacalai tesque) were used. Following several washes with 0.1% triton-X/PBS, cells were incubated with Alexa Fluor-conjugated secondary antibodies (Thermo Scientific) for 1 hr at RT. Following antibody treatment, cells were stained with DAPI (Sigma) to mark nuclei and were observed using a confocal laser scanning microscope.

Gene Expression & Western Blot analysis

For qRT-PCR total RNA was isolated and DNAse treated using the RNeasy mini kit (Qiagen), and reverse transcribed using Superscript III (Invitrogen). Gene expression was quantitated in quadriplucate using JumpStart SYBR green (Sigma) qPCR reagent and gene-specific primer sets on a QuantStudio 6 Flex Real-Time PCR System (Applied Biosystems), and normalised to *Gapdh* and *Arbp*. For western blots whole-cell extracts were prepared from WT or *Klf4* KO ESCs in lysis buffer composed of 50mM Tris-HCl (pH7.5), 0.15M NaCl, 0.1% SDS, 1% Triton X-100, 1% sodium deoxycholate and Complete-mini EDTA-free (Roche). After electrophoresis, proteins were transferred to nitrocellulose membranes. Membranes were incubated in blocking buffer containing skimmed milk and probed with Primary antibodies against KLF4 (goat IgG; R&D systems) and ACTIN (mouse IgG; sigma). Horseradish peroxidase-conjugated secondary antibodies against goat or mouse IgG were added (Dako). Blots were developed using SuperSignal West Pico Chemiluminescent Substrate (Thermo Scientific).

NGS Library Preparation

For RNA-seq, replicate ESC samples for each background were collected at independent timepoints (2 passages apart) following their culture in the specified media (total replicates per culture parameter n=4). 1μg of total RNA was extracted with RNeasy mini isolation kit (Qiagen), using on-column DNase steps. The poly-A fraction was isolated, fragmented and reverse transcribed into cDNA using the TruSeq RNA Library Prep kit v2.0, according to the manufacture's protocol. Double-stranded cDNA was end-repaired and Illumina adapters were ligated. Adapter-ligated DNA was purified with AMPure XP beads (Beckman Coulter) and amplified by PCR enrichment for 10 cycles. Quality control testing was carried using a TapeStation 2200 (Agilent). Individual samples were subsequently combined into multiplexed libraries and subjected to single-end 50bp sequencing on a HiSeq1500 (Illumina). For bisulfite sequencing libraries, genomic DNA was isolated with the Blood and Tissue kit (Qiagen) and sonicated to an average of 280bp in a cooled Bioruptor (Diagenode). 150ng of fragmented DNA was end-repaired and methylated indexed adapters were ligated using Ovation Ultralow Methyl-Seq Library system (Nugen). Bisulfite conversion was subsequently carried out using the EZ DNA Methylation Gold kit (Zymo Research), following the manufacture's recommendations. Lambda DNA was spiked in at 0.1% or 0.5% to track conversion efficiency. Bisulfite converted DNA was purified and amplified by PCR enrichment for 8 cycles using KAPA HiFi Uracil+ polymerase (Kapa bioscience). Individual samples were subsequently combined into multiplexed libraries and subjected to paired-end 125bp or single-end 100bp sequencing on a HiSeq1500 (Illumina). All data has been submitted to GEO under accession GSE98517.

Bioinformatics

RNA-seq analysis. Adapters were removed and reads were quality-trimmed using *TrimGalore*. Trimmed reads were aligned to the mouse reference genome (GRCm38/mm10) by using *TopHat2* (http://ccb.jhu.edu/software/tophat, version: 2.0.13) guided by ENSEMBL gene models. Raw counts per repeat regions and gene regions were obtained by *featureCounts.* Replicates were evaluated, counts were normalized and differential expression of transcripts was determined by the *R Bioconductor DESeq* package (www.bioconductor.org). Expression levels were further normalized by transcript length (per kB) where indicated. Transcript annotations in all bioinformatics analyses were based on Ensembl (Release 78). Principal component analysis was performed by singular value decomposition of the scaled expression values using the R svd() function (3D), and with the PCA function within *SeqMonk* software using log transformed merged transcript probes without length correction (2D). Gene Ontology (GO) term enrichment analyses were performed with the *Bioconductor topGO* package and the DAVID online tool v6.8 (https://david.ncifcrf.gov).

SOM analysis. All pairwise differentially expressed gene sets across the selected conditions were determined by DESeq (p value < 0.01, abs(log2FC) > 2). Self-organizing maps (SOMs) implemented by the *R kohonen()* package were trained to characterize distinct gene sets with similar transcriptional profiles in an unsupervised analysis. The size of the hexagonal SOM grid was set such that each node contained on average ~50 genes after classification.

*Repeat expression analysis***.** The mouse RepeatMasker annotation file (UCSC GRCm38/mm10) was downloaded from UCSC Tables; tRNA and rRNA repeat annotations were discarded. Trimmed reads were aligned to the mouse reference genome with *bowtie* (paramters: -m 1 –v 2 –best -strata) allowing for two mismatches and filtering uniquely mapping reads only. Raw counts per repeat regions and gene regions were obtained by *featureCounts* (parameter: -O) allowing for multiple feature assignments. Counts per repeat region were normalized by a factor between 0 and 1 obtained by the total number of counts in all genic regions divided by 10E+7. Normalized counts per retrotransposon family were pooled, and further normalized by the summed length of all individual repeat loci. Differential expression of families of retrotransposons and *p* values were estimated by the Bioconductor *DESeq* package. The fraction of active repeat loci per family was obtained by using an expression threshold of log2(normalized expression level) > 2 for each genomic repeat region.

Chimeric transcripts. Chimeric transcripts were obtained by extracting 50 nt reads that splice from repeat loci into genic exons based on the alignments generated by *TopHat2*. Repeat loci were required to be located 50 kB upstream of the transcriptional start sites of the largest annotated transcript per gene. i.e. potential splicing from intronic repeats to genic exons was not considered. A minimum of 2 independent spliced TE-gene chimeric reads was required to confirm the splicing event.

Whole genome bisulfite sequencing analysis. Bisulfite converted-reads were quality-trimmed with *Trim Galore*. Trimmed reads were then mapped to a computationally bisulfite-converted mouse reference genome (GRCm38/mm10) using *Bismark* (version: 0.7.12; parameter settings: *'-n 2 -l 40'*) tolerating two non-cytosine mismatches. Potential PCR duplicates were removed using *samtools rmdup*. CpG methylation calls were extracted from deduplicated mapping output using the Bismark methylation extractor in single-end mode. CpG methylation calls

were analysed using R and *SeqMonk* software. To calculate CpG methylation levels the genome was partitioned into consecutive 5-kb tiles or probes corresponding to defined genomic features covered by at least 5 CpGs, and percentage methylation was calculated using the bisulfite feature methylation pipeline in *SeqMonk*. Respective genomic features were defined using UCSC table browser annotations, *RepeatMasker* annotations or published datasets. Heatmaps were generated using the 'heatmap.2' function within R.\

Antibodies & Oligo Sequences

Antibodies

Antigen	Company	Clone number	Cat. number	Application
CD31	Biolegend	MEC13.3	557377	FACS
NANOG	eBioscience	eBioMLC-51	14-5761-80	IF
OTX2	Millipore		AB9566	IF
PAX6	Covance		PRB-278P	IF
BLIMP1	eBioscience	6D3	14-5963-82	IF
GATA4	Abcam	EPR4768	ab134057	IF
SOX17	R&D systems		AF1924	IF
KLF4	R&D systems		AF3158	IF
KLF4	Abcam		ab129473	WB
EGFP	Nacalai tesque	GF090R	04404-26	IF
HA	CST	6E2	2367S	WB
β-Actin	CST	8H10D10	3700T	WB

qRT-PCR

Gene	Forward	Reverse
Arbp	CAAAGCTGAAGCAAAGGAAGAG	AATTAAGCAGGCTGACTTGGTTG
Oct4	CCAATCAGCTTGGGCTAGAG	CTGGGAAAGGTGTCCCTGTA
Sox2	CATGAGAGCAAGTACTGGCAAG	CCAACGATATCAACCTGCATGG
Nanog	ACCTGAGCTATAAGCAGGTTAAGAC	GTGCTGAGCCCTTCTGAATCAGAC
Esg1	AAGGAGTGCTGAAGCTGGAGG	CAGCTTAACCTGCATCCAGGTC
Klf4	CGTCCCAGTCACAGTGGTAA	AAAAGAACAGCCACCCACAC
Blimp1	AAACGTGTGGGTACGACCTT	CCTTGAAACTTCACGGAGCC
Ap2g	TGAAGATGAAGCTGGGCTTT	TCCATTCTCTTCCGGTTCAG
Stella	AGGCTCGAAGGAAATGAGTTT	TCCTAATTCTTCCCGATTTTC
Nanos3	CACTACGGCCTAGGAGCTTGG	TGATCGCTGACAAGACTGTGG
Uhrf1	CCCCTCGCAACGGAAGAGCG	CGCCCACCACACACATGGCA
Gata4	AGGCACATGACCCATCACACA	AGAGGAAGGGAAGGCACCATG
Sox7	CCACAGTCCTTTGGCTGTCC	TACACGTGTCCAAGGGCAGA
Myc	TAACTCGAGGAGGAGCTGGA	GCCAAGGTTGTGAGGTTAGC
Eras	GTAGCTGTGGCTGCTCTGTAG	GATGTCTGTGGTAACTTGGTCG
Gbx2	GTGCCCAAAGGTAAACAGGA	AAATCAACCGACTGCTCTGC
Tfcp2l1	TGGCTACCACATCATCCTGA	GCTTGTCAGGTGAGACAGCA
Klf2	ACCAAGAGCTCGCACCTAAA	GTGGCACTGAAAGGGTCTGT
Tbx3	TTATTTCCAGGTCAGGAGATG	GGTCGTTTGAACCAAGTCCCT
Sox17	TTCTGTACACTTTAATGAGGCTGTTC	TTGTGGGAAGTGGGATCAAG
Dnmt3b	CTCGCAAGGTGTGGGCTTTTGTAAC	CTGGGCATCTGTCATCTTTGCACC

ChIP

**Gene**	**Forward**	**Reverse**
chr10:44,458,383 -44,458,520	GGGGGAGAAAAGAAAAGTTAAAA	CGACCTTGGTAAGGAACCAG
chr10:44,458,522 -44,458,721	CTCTCATGTCCACCCAGTCC	GCGGCCGTAGAAAAGGAG
chr10:44,458,713 -44,458,912	AGTGAGCGAGCGACTGACTA	GCGGCTGGTAGGAGTGAAT
chr10:44,459,053 -44,459,152	GGGGACTCCTCCTCAAAAGA	TGCATGTGCTGCCAAAATAC
chr10:44,392,037 -44,392,236	ACCCATCTTTGTCTGGGATG	AGCCCTGGAGGAGGAACTT
Oct3/4 DE (PC)	GACGGCAGATGCATAACAAA	AGGAAGGGCTAGGACGAGAG
RLP30 (NC)	#7015P in SimpleChIP Kit	

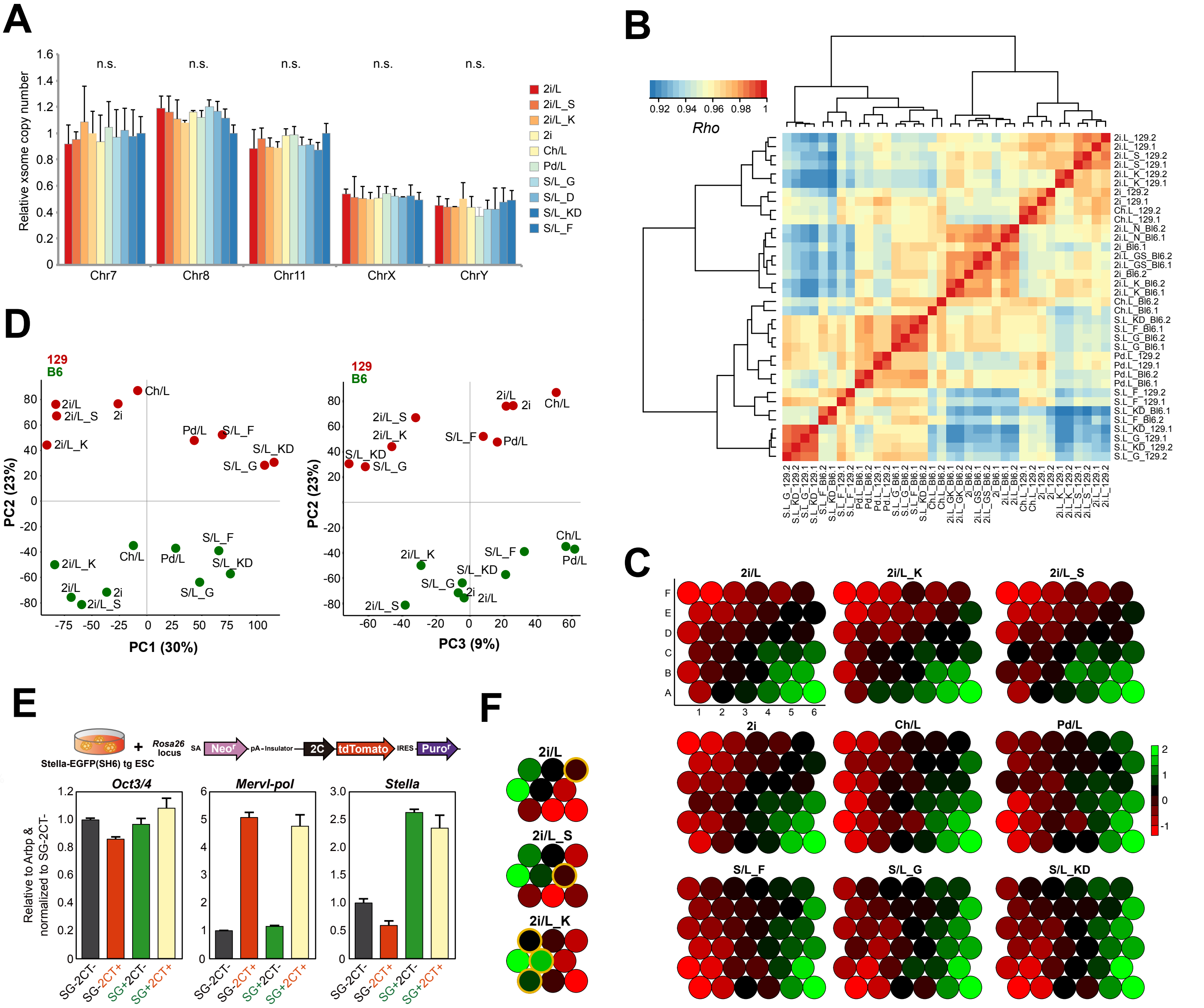

Figure S1. **Transcriptional characterisation of ESC states.** (A) Relative copy number of most commonly anueploid autosomes, and sex chromosomes, following transition to each ESC culture condition by qRT-PCR. (B) Correlation between individual replicates of RNA –seq. (C) Self-organising maps (SOM). Each circle represents the expression level of subset of all genes with similar co-expression dynamics in each ESC condition. The genes represented in position A2, for example, are highly expressed in 2i/L_K relative to other conditions. (D) Principal component analysis of ESC transcriptomes by culture condition and strain using an independent algorithm from Fig 1D (main article). (E) Expression level of indicated genes by qRT-PCR in *Stella*-GFP & 2C::tdTomato ESC, showing correlation between reporter and endogenous loci. (F) SOM comparing the addition of serum or KSR to naïve 2i/L culture conditions. Yellow circles represent genes subsets most significantly changing.

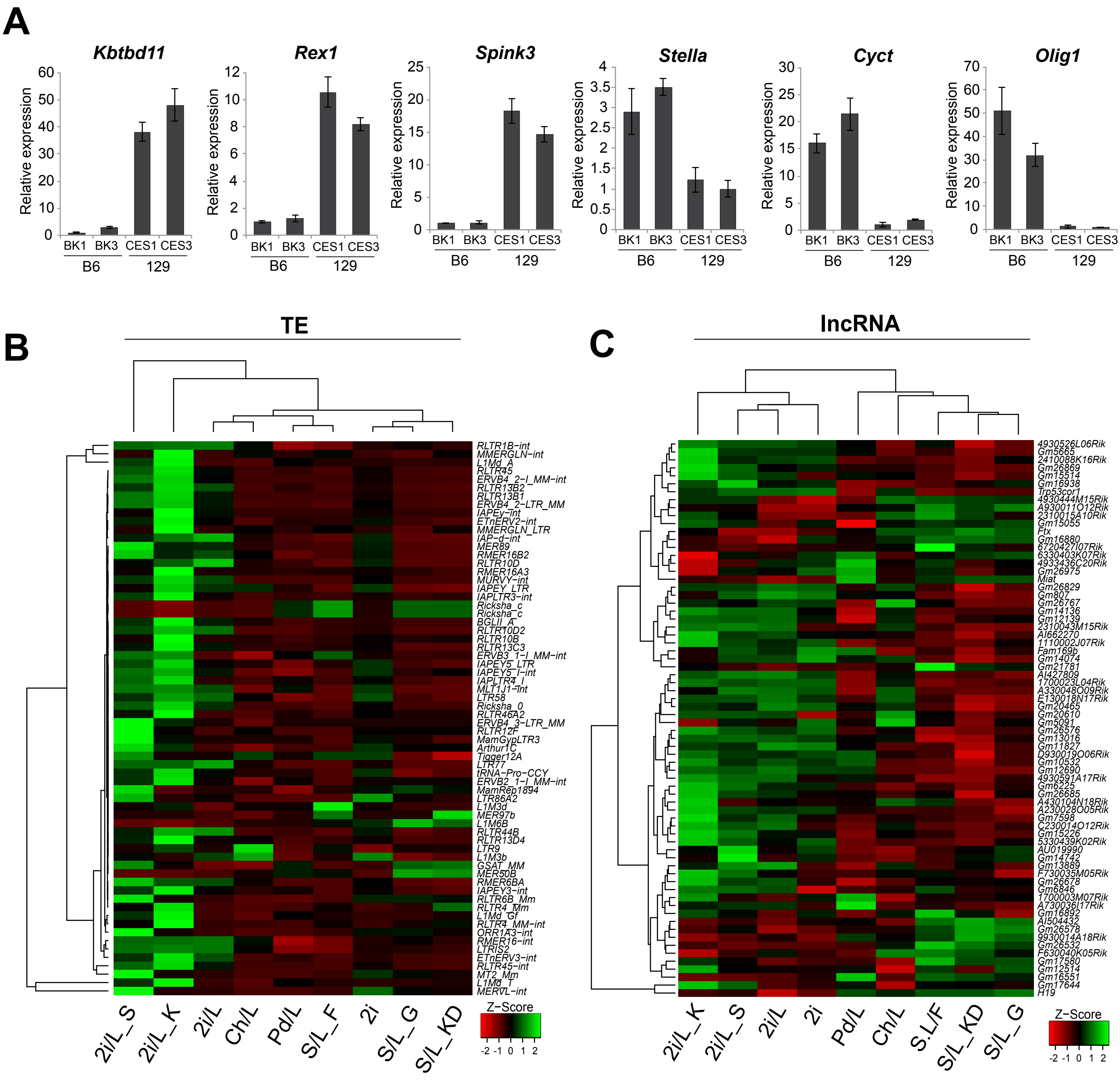

Figure S2. Expression of repetitive and non-coding elements. (A) Validation of differential expression between B6 and 129 ESC by qRT-PCR using independent lines from Fig 1. (B) Heatmap showing relative expression of transposable element (TE) classes identified as significantly differentially expressed between at least one pairwise comparison of culture conditions. (C) Heatmap showing relative expression of long noncoding RNAs (lncRNA) identified as significantly differentially expressed between at least one pairwise comparison of culture conditions. All analysis was performed on combined 129 and B6 datasets (n=4 per condition) to identify robust changes due to culture parameters.

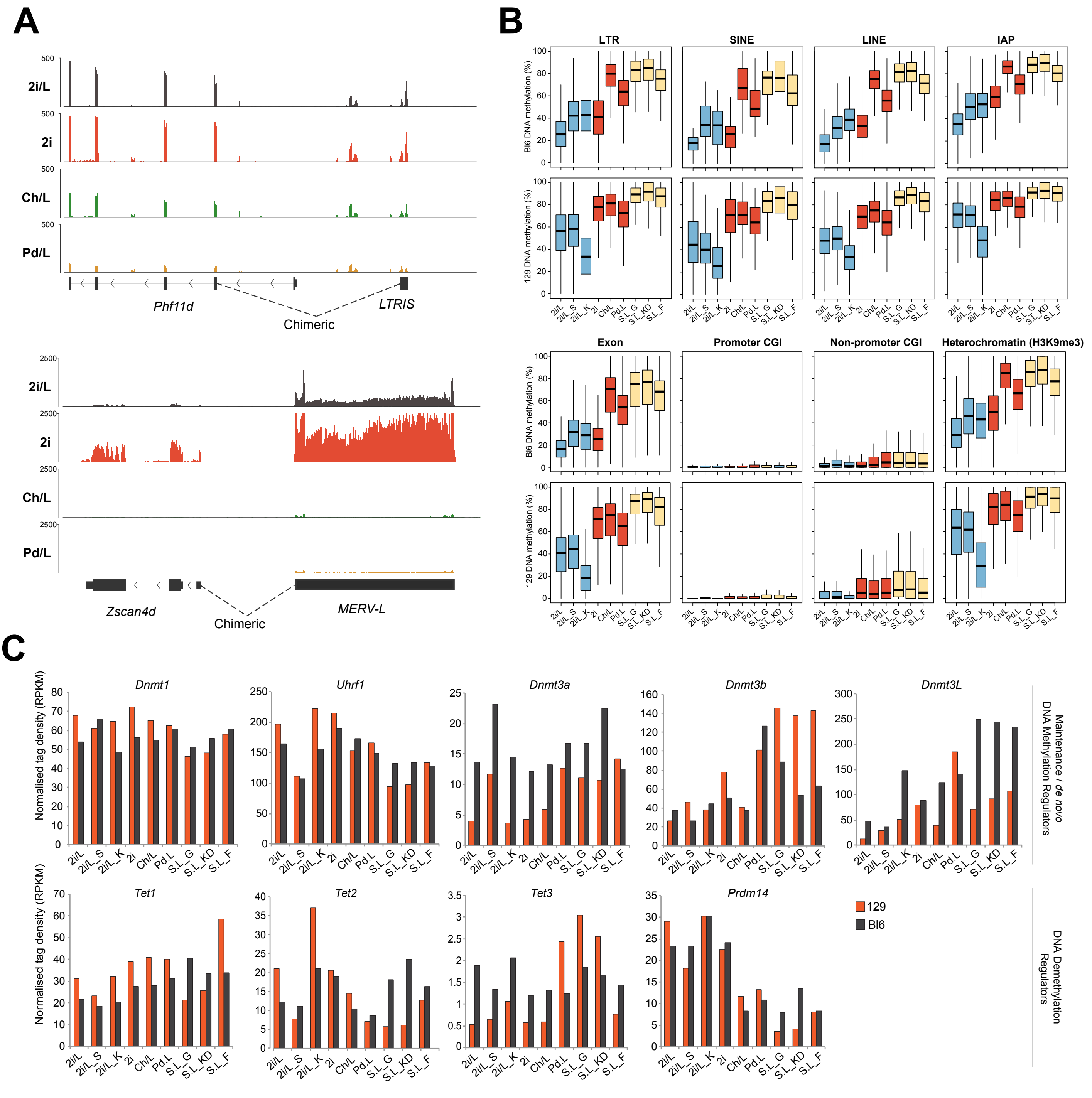

Figure S3. Chimeric and epigenomic analysis in alternative ESC states. (A) Genome view of informatically identified chimeric transcripts of *Phf11d* and *Zscan4d*, which are derived from an upstream LTR element in an ESC-state specific manner. (B) Boxplots showing median global DNA methylation levels across multiple repeat (upper) or genomic features (lower) in indicated ESC conditions and backgrounds. (C) Expresssion of multiple genes involved in DNA (de)methylation, in 129 and B6 ESC. The transcriptional levels of these genes do not definitively correlate with the global DNA methylation level across culture conditions and genetic background.

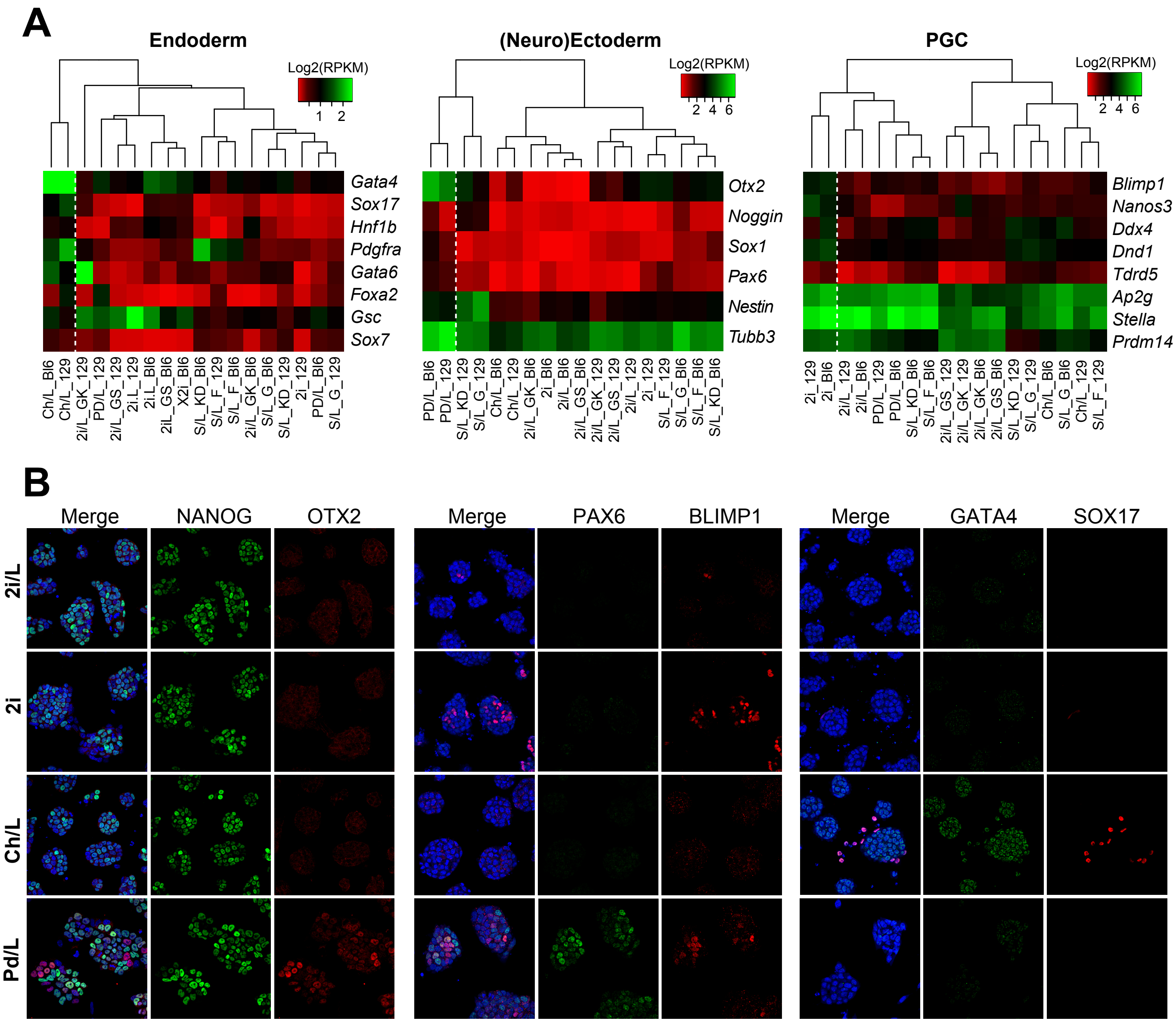

Condition	Sox17/Gata4	Pax6	Blimp1	Otx2
2i/L	0%	0%	1.6%	0%
2i	1.6%	0%	12.9%	0%
Ch/L	11.0%	0%	0%	0%
Pd/L	0%	26.7%	10.6%	37.6%

Figure S4. Activation of master lineage-regulators in naïve ESC culture conditions. (A) Heatmap showing absolute expression of key master regulators for endoderm, (neuro)ectorderm and primordial germ cells (PGC). Unbiased hierarchical clustering shows Pd/L, Ch/L and 2i, respectively, each segregate separately from all other conditions based on expression if these primary germ layer genes. (B) Immunofluorescence of master germ layer regulators confirms protein expression in only a specific culture condition, whilst remaining silenced in all others. Table shows quantification of strong-positive cells in each condition.

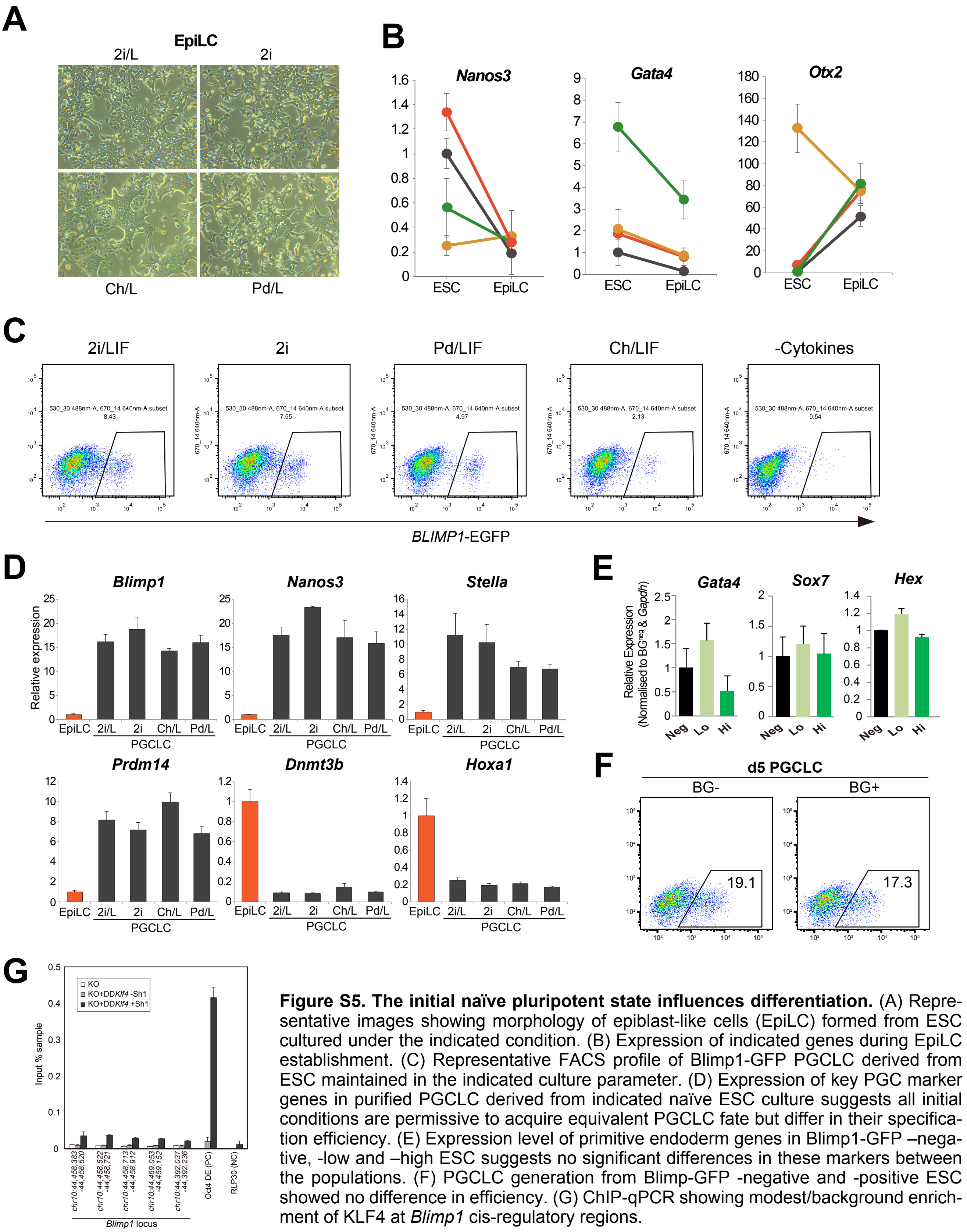

Figure S5. The initial naïve pluripotent state influences differentiation. (A) Representative images showing morphology of epiblast-like cells (EpiLC) formed from ESC cultured under the indicated condition. (B) Expression of indicated genes during EpiLC establishment. (C) Representative FACS profile of Blimp1-GFP PGCLC derived from ESC maintained in the indicated culture parameter. (D) Expression of key PGC marker genes in purified PGCLC derived from indicated naïve ESC culture suggests all initial conditions are permissive to acquire equivalent PGCLC fate but differ in their specification efficiency. (E) Expression level of primitive endoderm genes in Blimp1-GFP –negative, -low and –high ESC suggests no significant differences in these markers between the populations. (F) PGCLC generation from Blimp-GFP -negative and -positive ESC showed no difference in efficiency. (G) ChIP-qPCR showing modest/background enrichment of KLF4 at *Blimp1* cis-regulatory regions.
