# Supplementary material for: Activation of Lineage Regulators and Transposable Elements across a Pluripotent Spectrum
Source: Stem Cell Reports. 2017 Jun 9;8(6):1645–58. doi: 10.1016/j.stemcr.2017.05.014 (PMC5470235; doi:10.1016/j.stemcr.2017.05.014)
Supplement: Document S2. Article plus Supplemental Information [file mmc2.pdf]

Stem Cell Reports
Article
ISSCR
OPEN ACCESS

Activation of Lineage Regulators and Transposable Elements across a Pluripotent Spectrum

Jamie A. Hackett,[1,2,3,4] Toshihiro Kobayashi,[1,2,4] Sabine Dietmann,[1,2] and M. Azim Surani[1,2,*]
[1]Wellcome Trust/Cancer Research UK Gurdon Institute, University of Cambridge, Tennis Court Road, Cambridge CB2 1QN, UK
[2]Department of Physiology, Development and Neuroscience, University of Cambridge, Downing Street, Cambridge CB2 3DY, UK
[3]European Molecular Biology Laboratory (EMBL) - Monterotondo, via Ramarini 32, 00015, Rome, Italy
[4]Co-first author
*Correspondence: a.surani@gurdon.cam.ac.uk
http://dx.doi.org/10.1016/j.stemcr.2017.05.014

SUMMARY

Embryonic stem cells (ESCs) are characterized by the pluripotent capacity to generate all embryonic lineages. Here, we show that ESCs can occupy a spectrum of distinct transcriptional and epigenetic states in response to varied extrinsic conditions. This spectrum broadly corresponds to a developmental continuum of pluripotency and is coupled with a gradient of increasing global DNA methylation. Each pluripotent state is linked with activation of distinct classes of transposable elements (TEs), which in turn influence ESCs through generating chimeric transcripts. Moreover, varied ESC culture parameters differentially license heterogeneous activation of master lineage regulators, including *Sox1, Gata4*, or *Blimp1,* and influence differentiation. Activation of *Blimp1* is prevalent in 2i (without LIF) conditions, and marks a dynamic primordial germ cell (PGC)-like sub-state that is directly repressed by *Klf4* downstream of LIF/STAT3 signaling. Thus, extrinsic cues establish a spectrum of pluripotent states, in part by modulating sub-populations, as well as directing the transcriptome, epigenome, and TE.

INTRODUCTION

Pluripotency is a transient state established during mammalian preimplantation development, and is characterized by the capacity to give rise to all fetal lineages. The pluripotent state can be propagated indefinitely through derivation of embryonic stem cells (ESCs) or via reprogramming strategies (De Los Angeles et al., 2015). Mouse ESCs are considered to exhibit naive pluripotency, which reflects their ability to contribute to all embryonic lineages upon re-introduction into a blastocyst, as well as other key hallmarks such as a derestricted epigenome and two active X chromosomes in female cells (Hackett and Surani, 2014). The preservation of naive pluripotency in ESCs is underpinned by expression of a network of auto-regulatory transcription factors, including *Oct4*, *Sox2*, *Nanog*, and *Tfcp2l1*, which are themselves sustained by extrinsic signaling cues (Dunn et al., 2014). An alternative, and possibly more developmentally advanced state of pluripotency, can also be propagated, as exemplified by epiblast stem cells (EpiSCs) and human ESCs (hESCs) in conventional culture conditions. These are typically classified as being in a *primed* pluripotent state that is poised to initiate lineage decisions (Nichols and Smith, 2009).

A broader array of pluripotent states is, however, likely. For example, culture using inhibitors of MAPK signaling and GSK3β (termed 2i) together with LIF (2i/L), render mouse ESCs in a relatively uniform naive state that is molecularly and epigenetically distinct from ESCs in conventional serum/LIF medium (Ying et al., 2008; Marks et al., 2012; Leitch et al., 2013). Any two of the 2i/L components in various combinations also yield naive ESCs, which may occupy distinct phases of pluripotency (Wray et al., 2010). Multiple other pluripotent conformations could also arise depending on the derivation strategy, available metabolites, and the precise signaling regime supplied, which may reflect distinct spatial or temporal identities (Blaschke et al., 2013; Tonge et al., 2014; Wu et al., 2015; Huang et al., 2014; Irie et al., 2015; Weinberger et al., 2016). Collectively, this suggests that a naive/primed duality model may not capture the broad complexity of pluripotency in vitro and possibly in vivo. Instead a graded spectrum of pluripotent states may emerge that exhibit distinct molecular and functional properties (Hackett and Surani, 2014; Wu and Izpisua Belmonte, 2015).

Heterogeneity in pluripotent stem cell populations is also prevalent, in part driven by dynamic sub-states. ESCs in conventional serum/LIF conditions interconvert between several configurations that include naive and primed sub-populations (Torres-Padilla and Chambers, 2014; Klein et al., 2015). Naive ESCs cultured in 2i/LIF are also apparently composed of sub-populations (Kolodziejczyk et al., 2015; Morgani et al., 2013), while EpiSCs and hESCs are also known to be highly heterogeneous (Cahan and Daley, 2013). This implies that dynamic heterogeneity may be a fundamental feature of pluripotent stem cells. Indeed, mouse ESCs continually transit through a *Zscan4*$^+$ sub-state that promotes transient DNA demethylation and telomere rejuvenation, with the latter being essential for sustained pluripotent viability (Zalzman et al., 2010;

This is an open access article under the CC BY license (http://creativecommons.org/licenses/by/4.0/).

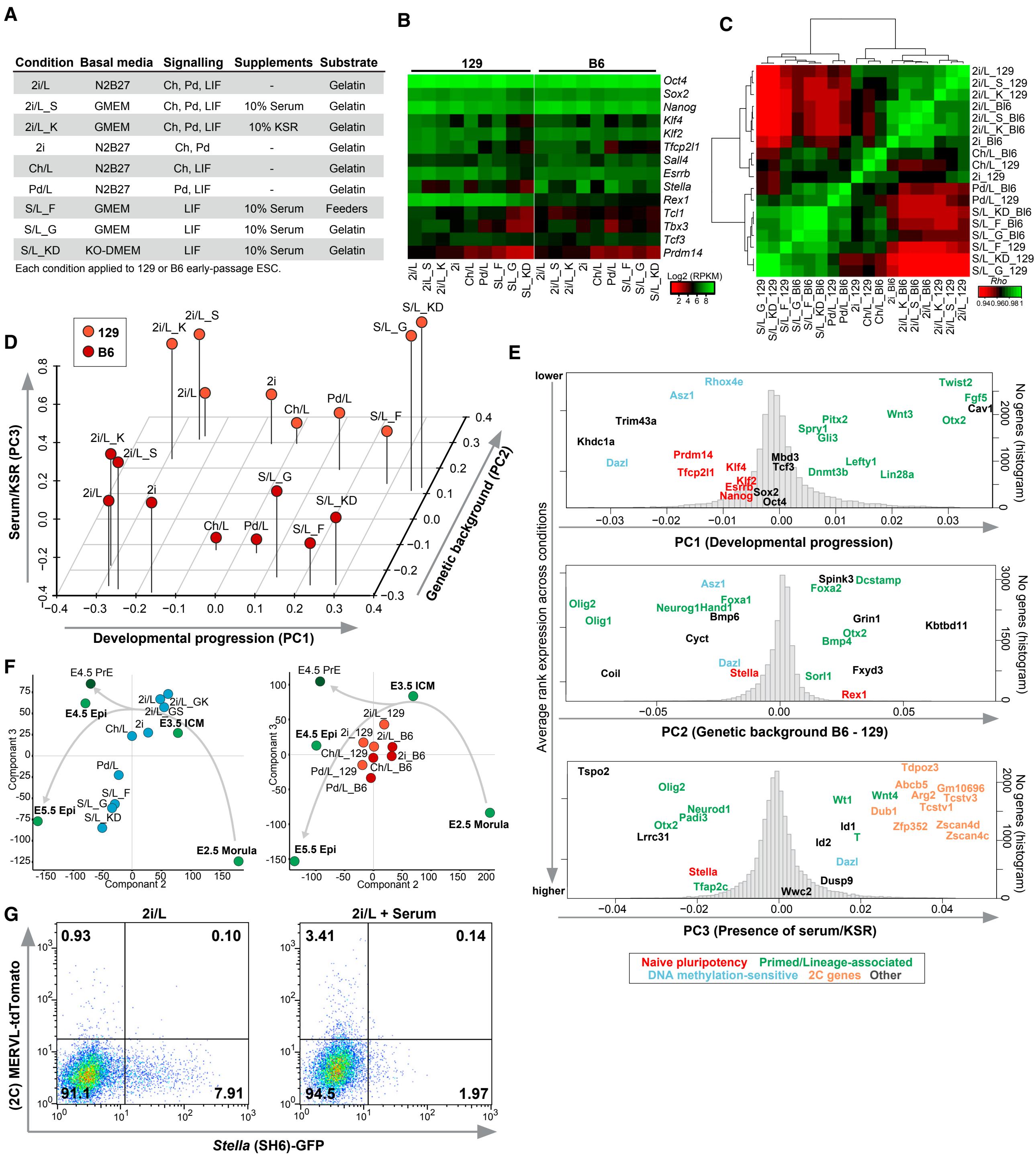

Figure 1. A Transcriptional Spectrum of Pluripotent States

(A) ESC culture conditions assayed in this study.

(B) Heatmap showing expression of key pluripotency genes by RNA-seq.

(C) Global transcriptional correlation between all genetic backgrounds and culture parameters.

(D) Principal-component analysis (PCA) showing the relationship between pluripotent ESC states.

(legend continued on next page)

Eckersley-Maslin et al., 2016). Passing through sub-states can therefore imprint a significant memory, implying that the prevalence of sub-populations could have broad functional implications for the whole ESC population.

It has recently emerged that successive stages of early mammalian development are linked with expression of distinct classes of transposable element (TE) (Goke et al., 2015). For example, MERVL elements and their cognate MT2 LTR become active specifically at the 2-cell (2C) stage in mice, while HERVK is active from the 8-cell stage in human embryos (Peaston et al., 2004; Grow et al., 2015). These elements can significantly influence expression of nearby genes. For example, TE can act as co-opted promoters that splice to downstream genes thereby generating "chimeric transcripts" (Macfarlan et al., 2012). In addition, TEs can affect gene expression through promoting open chromatin configurations, production of long noncoding RNAs (lncRNA), or by acting as enhancers (Thompson et al., 2016). The impact of TEs may be particularly prevalent in pluripotent cells, since TEs are under selective pressure to be active in pluripotent or germline phases in order to propagate transgenerationally (Bourque et al., 2008). Indeed, HERVH elements have a key role in contributing to the pluripotency network in hESCs (Wang et al., 2014). Thus, TEs represent a relatively unexplored regulatory source for the establishment and control of alternate pluripotent states.

Here, we identify a spectrum of ESC states and characterize the distinct transcriptional networks and epigenome at each node. Distinct classes of TEs are active between pluripotent conformations and influence the emergent transcriptome. Strikingly, some naive culture conditions license dynamic activation of master regulators for a specific primary germ layer (endoderm or ectoderm) or primordial germ cells (PGCs). Mechanistically, we identify *Klf4* and LIF/STAT3 as the key regulators of a *Blimp1*$^{+}$ PGC-like ESC state. Overall we report that ESC populations can occupy a continuum of transcriptional states, in part through accessing sub-states under certain culture parameters.

RESULTS

Distinct Transcriptional States of Pluripotency

We initially sought to define the transcriptional variation across a broad range of pluripotent ESC states, with the expectation that any underlying differences may influence differentiation, particularly toward PGC fate. We selected nine culture parameters capable of supporting naive pluripotency, as judged by chimera contribution, and transited male (XY) low-passage (<p13) 129X1/SvJ (129) or C57BL/6J (B6) ESCs into each condition for ≥ 5 passages. The culture parameters were designed to test the influence of multiple extrinsic factors including combinations of GSK3β inhibition/WNT activation (CHIR99021, hereafter Ch), MEK inhibition (PD0325901, hereafter Pd), STAT3 activity (LIF), BMP/undefined signaling (serum), vitamin C/undefined signaling (knockout serum replacement, hereafter KSR), basal medium, feeders, and genetic background, on the overall state of pluripotency (Figure 1A). We observed no overt karyotype alterations during transition to each condition, as judged by indicator chromosomes (Figure S1A; D'Hulst et al., 2013).

RNA sequencing (RNA-seq) revealed that ESCs in each condition expressed high levels of key pluripotency genes *Oct4, Sox2*, and *Nanog,* supporting their pluripotent status (Figure 1B). Some naive pluripotency genes varied among culture parameters, however, presumably reflecting the presence or absence of their direct signaling regulators. For example, *Klf4* is downregulated in 2i conditions that lack LIF, consistent with *Klf4* being a direct LIF/STAT3 target (Hall et al., 2009), while *Tfcp2l1* is repressed in Pd/L, likely owing to attenuated WNT transduction (Ye et al., 2013). Pluripotent markers *Stella* and *Rex1* primarily exhibited reciprocal background-dependent expression; with *Rex1* elevated in 129 ESCs and *Stella* upregulated in B6 ESCs (Figure 1B). In general, ESCs in conventional serum/LIF (S/L) conditions expressed lower overall levels of naive markers, particularly when feeder free. Thus, while the broad network of naive pluripotency genes is comparable among ESC conditions, the precise transcriptional levels of each component vary downstream of culture parameters and genetic background.

The correlation of global gene expression patterns revealed two broad clusters; a response to combinations of 2i/L components, or ESCs under S/L and Pd/L conditions (Figures 1C and S1B). We found 3,048 genes exhibited robust differential expression (Log2(FC) > 2; adjusted $p < 0.01$) in pairwise comparisons between all states, and these assembled into distinct co-expression clusters associated with specific gene ontologies (Figure S1C). In addition, 136 and 82 genes are robustly linked with 129- or B6-specific expression, respectively, which we confirmed using multiple independent 129 and B6 ESC lines (Figure S2A).

(E) Loadings for PCA from (D) showing representative highly weighted genes that drive separation along each component. The histogram shows the distribution of all gene weightings.
(F) PCA analysis showing in vivo pluripotent stages and the full ESC spectrum (left) or defined ESC states separated by genetic background (right).
(G) Fluorescence-activated cell sorting (FACS) plot showing the response of *Stella*-GFP and *MERVL*-tdTomato (2C) ESCs in 2i/L to serum.

To further investigate the relationship between each pluripotent condition we applied principal-component analysis (PCA). Strikingly, we observed a clear separation of ESC states along the first component (PC1) that appeared to reflect an ordering of developmental progression; ranging from 2i/L (with or without serum/KSR) through to feeder-free S/L (Figures 1D and S1D). By contrast PC2 separated ESCs according to genetic background (129 versus B6), while PC3 apparently segregated ESCs cultured in the presence or absence of serum/KSR, irrespective of other signaling influences (Figure 1D). To validate these interpretations, we examined the gene loadings along each principal component to determine the key driver genes that separate ESC states. Notably, negative PC1 values were strongly weighted by naive-associated genes, and in particular *Tfcp2l1* and *Prdm14*. In contrast, positive PC1 scores were driven by primed and early developmental genes including *Fgf5, Otx2*, and *Lefty1* (Figure 1E). This is consistent with separation along PC1 reflecting a spectrum of pluripotent ESC states that range from most naive to most developmentally progressed, with conditions such as Ch/L establishing intermediary states of naive pluripotency.

To investigate how this apparent pluripotent spectrum relates to embryonic ontogeny, we compared the ESC transcriptomes with publically available in vivo developmental stages (Boroviak et al., 2015). The first component here reflected technical differences but the analysis still recapitulated the continuum of ESC states, and, strikingly, they broadly correspond to developmental progression of the pluripotent epiblast lineage (Figure 1F). Specifically, the most naive ESC states cluster closest with embryonic day 3.5 (E3.5) inner cell mass, whereas ESC states predicted to be further along the pluripotent spectrum become progressively more comparable with E4.5 and, to some degree, E5.5 epiblasts (Figure 1F).

We next examined the other principal components that separate ESC states and observed that gene loading along PC2, which delineates genetic background, exhibited no significant gene class enrichment, albeit some neuroectodermal genes such as *Olig1* are linked with B6 ESCs (Figure 1E). This implies that, although background has significant influence on the precise ESC transcriptome, no specific gene category predominates. Finally, gene loadings for PC3 revealed strong weighting for BMP targets *Id1* and *Id2*, along with mesendoderm genes, consistent with separation of ESC transcriptomes according to the presence of BMP-rich serum or KSR. However notably, the most significant PC3 weightings (nine out of ten top genes) correspond to genes preferentially expressed in 2-cell embryos, including *Zscan4* and *Tcstv3,* suggesting the 2C program is the single most affected pathway downstream of serum/KSR supplementation (Figure 1E).

To further investigate this and to test the PCA predictions we generated ESCs carrying dual reporters for 2C gene activity and *Stella,* which are expected to respond reciprocally to serum (see PC3) (Figure 1E). Consistently, we found that addition of serum to 2i/L reduced the fraction of *Stella*-positive ESCs by 4.0-fold, while concurrently increasing the fraction of 2C-positive ESCs by 3.7-fold (Figures 1G and S1E). In summary, we identify a spectrum of multiple naive pluripotent states that emerge in response to distinct combinations of extrinsic signaling cues. This spectrum appears to correspond to a developmental progression of pluripotency. The emergent ESC states are further dispersed depending on genetic background and/or additional supplements, such as serum or KSR. Moreover, the spectrum may in part reflect different sub-population identities and frequencies between culture parameters, as exemplified by 2C and *Stella*-positive ESCs.

Retrotransposon Activation Is Linked with Pluripotent State

Stage-specific activation of distinct TEs has been observed during successive stages of early embryonic development (Goke et al., 2015). To investigate whether each state along the ESC pluripotent spectrum is also linked with a specific signature of TE activity, we examined differentially expressed repeat families. We found 64 significantly altered families (total n = 1,110) in pairwise comparisons, of which 51 belonged to the LTR class of retrotransposons, including MERVL, MT2, and IAPLTR3 (Figures 2A and S2B). Several TE families are preferentially active in a specific ESC condition, for example LTR9 and L1M3d in Ch/L and S/L_F, respectively. Interestingly, PCA analysis of TE expression ordered the samples into a highly comparable arrangement as gene-based PCA, apparently recapitulating the pluripotent continuum (Figure 2B).

Examining loading of retrotransposons in the PCA revealed that families including IAPEy-int and RLTR45 strongly weighted the most naive-like ESC states (2i/L, 2i), whereas higher expression of MER50B and the LINE family L1M6B was linked with more developmentally progressed pluripotent identities (Figure 2C). The strong weighting of IAPEy-int is noteworthy as this family shares closest homology with HERVK elements, which are implicated in influencing human pluripotent cells (Grow et al., 2015). We additionally found 70 differentially expressed lncRNA between ESC conformations, many of which originate at TEs (Figure S2C). Among these the lncRNA *Miat,* which is involved in a feedback loop with pluripotency-associated factors (Sheik Mohamed et al., 2010), is progressively upregulated in each pluripotent state along the spectrum (Figure 2D).

Next, we asked whether activation of distinct sets of TE is directly linked with altered gene expression. We

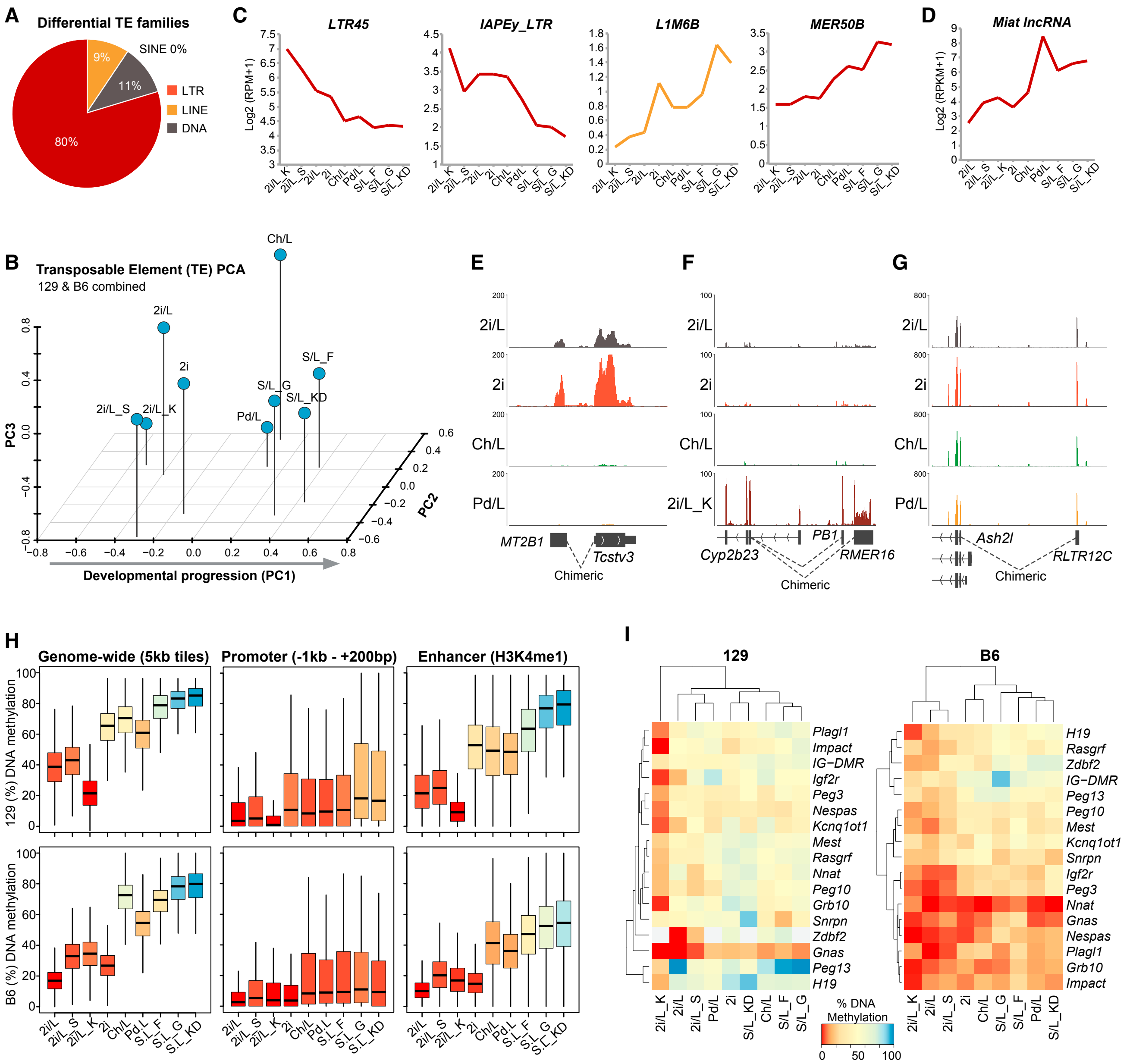

Figure 2. Transposable Element Activation across the Pluripotent Spectrum

(A) Relative proportion of differentially expressed transposable element (TE) families between pairwise comparisons of all conditions.

(B) PCA showing the relationship between pluripotent states based on differential TE activation.

(C) Expression of selected LTR and LINE elements across the pluripotent continuum.

(D) Expression of the lncRNA *Miat*.

(E–G) Genome view showing RNA-seq tracks of detected chimeric transcripts that originate at an upstream LTR and splice to (E) *Tcstv3*, (F) *Cyp2b23*, and (G) *Ash2l*.

(H) Boxplots showing the median global DNA methylation (5-mC) level by bisulfite sequencing across various genomic features in the indicated culture condition and genetic background.

(I) Heatmap showing DNA methylation level at genomic imprints.

initially noted that between defined conditions (serum-free), 2i ESCs preferentially activated MT2 and MERVL elements, as well as many 2C-associated genes that can derive from such repeats as chimeric transcripts. Among these, expression of the telomere regulators *Tcstv3* and *Zscan4*, and the metabolism gene *Arg2*, is highly elevated in 2i.

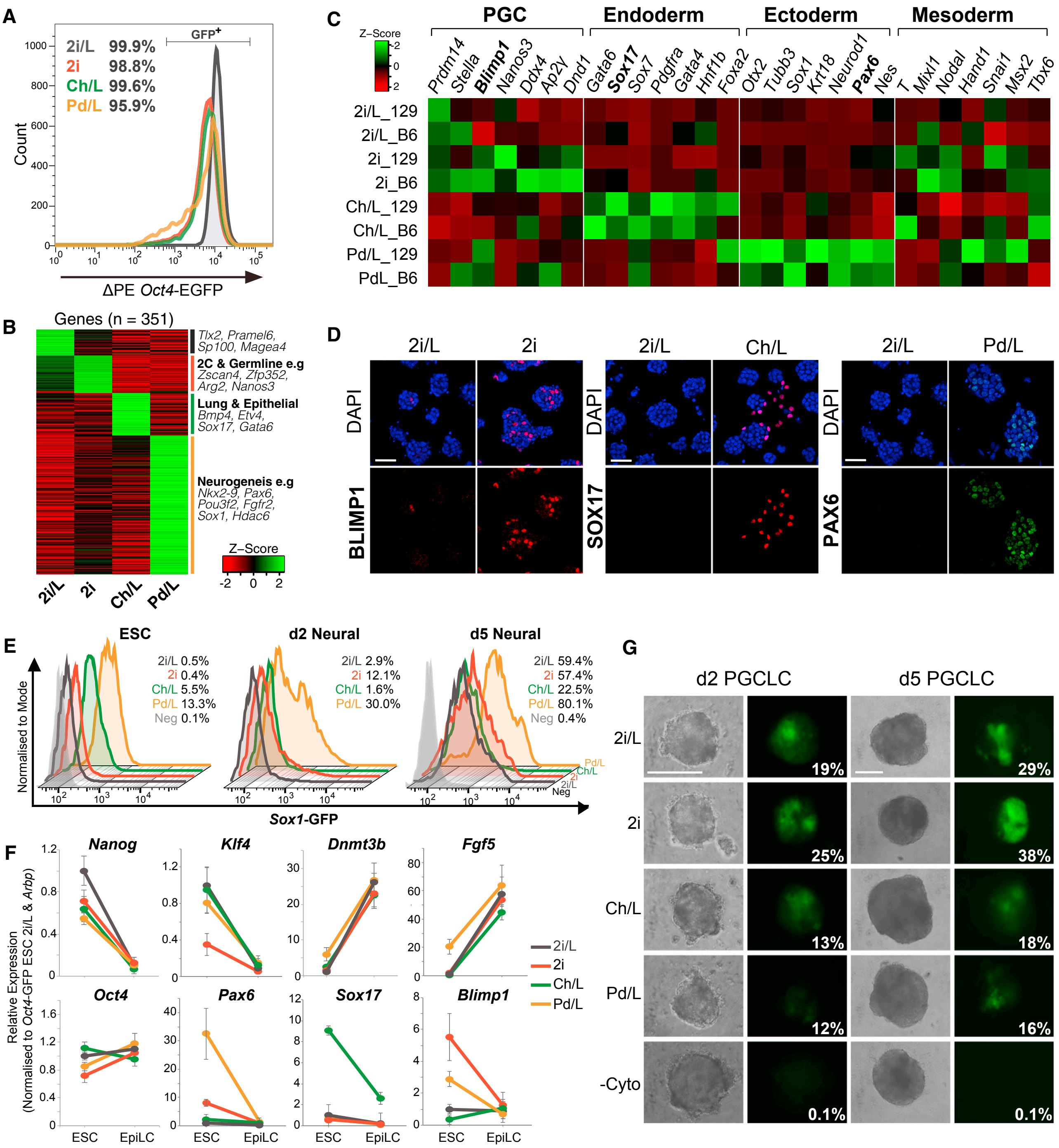

Figure 3. Distinct Naive ESC Conditions Activate Master Lineage Regulators

(A) FACS analysis showing near-uniform activation of the ΔPE *Oct4*-GFP naive pluripotency reporter in ESCs under defined conditions.

(B) Heatmap of differentially expressed genes in pairwise comparisons between naive ESC states. Selected genes from pathways enriched by gene ontology are shown.

(C) Heatmap showing expression of master regulator genes for each of the three primary germ layers and the germline (PGC).

(D) Immunofluorescence staining for master regulators of the germline (BLIMP1), endoderm (SOX17), and neuroectoderm (PAX6) in defined ESC conditions. Scale bars, 50 μm.

(E) FACS plots showing *Sox1*-GFP activation upon neural induction from indicated ESC conditions.

(legend continued on next page)

Analyzing spliced RNA-seq reads revealed that gene upregulation in 2i is often a direct consequence of activation of upstream MERVL and MT2 elements, which initiate chimeric transcripts (Figure 2E).

Using our RNA-seq datasets to detect additional spliced junctions, we identified a further 637 chimeric transcripts across the pluripotent conditions, which derive from activated TEs upstream of the first annotated exon. Many of these are active or repressed in only a subset of pluripotent states. For example an LTRIS2 element upstream of *Phf11d* is responsible for a chimeric transcript, but this TE is preferentially repressed in Pd/L ESCs. As a consequence *Phf11d* mRNA is significantly downregulated in the Pd/L pluripotent conformation (Figure S3A). Similarly, activation of adjacent EtnERV2, RMER16, and PD1D10 elements upon KSR addition drives strong expression of the downstream *Cyp2b23* gene (>30-fold transcriptional upregulation) specifically in 2i/L_K ESCs (Figure 2F). The potential significance of such TE-based regulatory mechanisms is underscored by our observation that a chimeric transcript derived from an RLTR12C element drives primary expression of the essential pluripotency gene *Ash2l* in ESCs (Wan et al., 2012; Figure 2G). Other key genes including *Grb2* and *Zfp640* also appear to be at least partly expressed from an upstream TE (data not shown). Taken together our data imply that each state of naive pluripotency is associated with a distinct repertoire of transcriptionally active retrotransposons, particularly among the LTR class. In some cases this is directly responsible for modulating gene transcription through chimeric transcripts. Notably, this links altered retrotransposon activity and overall ESC state.

DNA Methylation across the Pluripotent Spectrum

Global DNA hypomethylation is intimately associated with naive pluripotency, whereas increasing DNA methylation (5-methylcytosine, 5-mC) levels are generally linked with primed pluripotency and lineage commitment (Leitch et al., 2013). We used shallow-coverage whole-genome bisulfite sequencing to investigate 5-mC across the pluripotent spectrum. Genome-wide 5-mC levels were lowest in 2i/L (18%–37%) and most elevated in S/L_KD (78%–83%) ESCs, consistent with previous reports (Habibi et al., 2013; Ficz et al., 2013; Hackett et al., 2013a). However rather than falling into binary hypo- or hypermethylated status, other pluripotent states exhibited a gradient of progressive global 5-mC, with Pd/L (53%–61%) and Ch/L (69%–75%) associated with intermediate DNA methylation levels, for example (Figure 2H). Thus, similar to the continuum of transcriptional states, ESCs acquire a spectrum of epigenetic states.

The trend of DNA methylation was preserved across distinct genomic features such as promoters, enhancers, and repetitive elements (Figures 2H and S3B). An exception, however, is genomic imprints. These were relatively stable across most conditions (particularly in 129 background), but exhibited atrophy in 2i/L and erasure in 2i/L+KSR culture (Figure 2I). This latter observation may reflect the presence of vitamin C in KSR, which directly enhances TET activity, previously linked with erasure of imprints (Yamaguchi et al., 2013; Hackett et al., 2013b; Blaschke et al., 2013; Piccolo et al., 2013). In summary, the 5-mC epigenome is established across a wide spectrum in ESCs, ranging from hypo- to hypermethylated, with the precise level correlated to the developmental progression of the underlying pluripotent transcriptome and, to some extent, the presence of MEK inhibitor (Pd). The transcriptional level of genes known to influence DNA methylation levels, such as *Dnmt3a, Uhrf1, Tet1*, and *Prdm14,* did not definitively correlate with global 5-mC levels, however (Figure S3C). This may indicate that the overall epigenetic state in ESCs is regulated by a complex interplay between gene expression, available metabolites, and post-transcriptional control, such as recently shown for UHRF1 (von Meyenn et al., 2016).

Naive ESC Populations Exhibit Lineage-Associated Co-activation

To investigate the pluripotent spectrum in more detail, we selected four defined (serum-free) conditions (2i/L, 2i, Ch/L, and Pd/L) associated with a clear naive signature and robust chimera contribution (Dunn et al., 2014). We confirmed naive status by using the ΔPE *Oct4*-GFP reporter, which marks activation of the naive-specific distal *Oct4* enhancer in ESCs. All states exhibited a single peak of GFP, implying relatively uniform naiveté among these populations (Figure 3A). To examine any underlying molecular differences between the naive states, we identified 1,056 differentially expressed genes (Log2(FC) > 2; $p < 0.01$) in all pairwise comparisons. These genes were primarily linked with differential metabolic processes, but surprisingly also included several markers of alternate lineage fates. Indeed, gene ontology analysis of genes upregulated uniquely in only one condition suggested activation of divergent germ layer programs, such as neurogenesis-related processes in Pd/L (Figure 3B).

(F) qRT-PCR showing expression of indicated genes in ΔPE *Oct4*-GFP ESCs and upon induction of epiblast-like cells (EpiLCs) for 2 days. n = 3 biological replicates each assayed in technical triplicate; error bars, SEM.
(G) Representative examples of primordial germ cell (PGC)-like cell (PGCLC) induction from ancestral ESC conditions. Shown is the percentage of PGCLCs as determined by FACS. Scale bars, 200 μm.

To explore this further we inspected expression of master regulators for the three primary germ layers; endoderm, mesoderm, and ectoderm, as well as the germline, among the four naive states. Remarkably, we found that pluripotent ESCs in Pd/L strongly and specifically co-expressed multiple master (neuro)ectoderm genes such as *Sox1, Pax6*, and *NeuroD1*, but not markers of other lineages, in both 129 and B6 backgrounds (Figure 3C). By contrast, Ch/L ESCs upregulated master regulators for endoderm specification including *Sox17, Gata4*, and *Gata6,* as well as definitive endoderm markers *FoxA2* and *Hnf1b*. Finally, 2i conditions exhibited a clear PGC signature, uniquely co-activating the three key PGC specification genes *Blimp1, Prdm14*, and *Ap2γ*, and some primitive streak/mesodermal genes such as *Mixl1*. Unsupervised hierarchical clustering further revealed that 2i, Ch/L, and Pd/L each segregate separately from all other pluripotent conditions based only on expression of germline, endoderm, and neuroectoderm master regulators, respectively (Figure S4A). This highlights their exclusive overall state with respect to lineage-associated expression. Notably, however, master primary germ layer genes were near undetectable in 2i/L ESCs (Figure 3C).

We next sought confirmation for activation at the protein level by immunostaining. This revealed robust detection of the key germline-determinant BLIMP1 in 2i, endoderm regulators SOX17 and GATA4 in Ch/L, and neuroectoderm regulators PAX6 and OTX2 in Pd/L, but near-undetectable levels in reciprocal conditions (Figures 3D and S4B). NANOG was strongly detected under all parameters. In general, lineage-associated expression was heterogeneous, possibly reflecting dynamic expression. Using the Fucci reporter, we also noted altered cell-cycle dynamics between pluripotent states, which can influence lineage-bias in stem cells (data not shown) (Pauklin and Vallier, 2013). Overall, the tested pluripotent parameters appear to differentially license activation of lineage-specific master regulators, with a subset of ESCs shuttling into positive state.

To investigate whether lineage-associated expression influences cell fate, we seeded ESCs in N2B27 medium without cytokines, which preferentially induces neuroectoderm specification, but is also permissive for other lineages (Ying et al., 2003). Using *Sox1*-GFP ESCs that report on acquisition of neuroectoderm fate, we observed that cells from the ancestral Pd/L condition activated GFP both earlier and with greater maximal efficiency (>80%) than ESCs from other initial parameters. Moreover Ch/L maintained ESCs, which exhibited some activation of endoderm regulators, were relatively resistant to acquiring a *Sox1*-GFP$^+$ fate (18%–31%) (Figure 3E).

We next asked whether an alternative strategy of differentiation is also influenced by initial pluripotent state by inducing PGC fate. PGC specification proceeds through induction of naive ESCs into epiblast-like cells (EpiLCs), which closely parallel post-implantation epiblast cells, and subsequently specification of PGC-like cells (PGCLCs) in embryoids (Hayashi et al., 2011). All examined ESC states formed morphologically equivalent EpiLCs, which expressed comparable levels of "primed" markers such as *Fgf5* and *Dnmt3b*, while concurrently repressing naive genes including *Nanog* and *Klf4* (Figures 3F, S5A, and S5B). The differential expression of most lineage regulators also equalized during EpiLC induction. Nonetheless, we observed a marked difference in PGCLC specification from EpiLCs depending on the predecessor condition of ESCs.

Specifically, PGCLCs were specified with high efficiency from ancestral 2i/L ESCs and, in particular, from 2i ESCs. In contrast, PGCLC specification from initial Ch/L and Pd/L states was significantly impaired, as judged by detection of ΔPE *Oct4*-GFP and *Blimp1*-GFP (Figures 3G and S5C). PGCLCs derived from all ESC culture parameters exhibited appropriate germline gene expression signatures, suggesting that they all acquire PGC fate, but differ in their specification efficiency (Figure S5D). Thus, the initial pluripotent ESC condition establishes an enduring memory that affects subsequent differentiation potential, despite an apparently normalizing intermediate EpiLC step. Collectively, these analyses imply that the initial pluripotent parameters can influence the rate and efficiency of directed differentiation toward distinct lineages. This appears to be partly correlated with the differential licensing of lineage-associated programs between ESC conditions, albeit other factors also likely contribute.

ESCs Transit through a Blimp1$^+$ PGC-like State

We next sought to investigate the nature of lineage-associated programs in naive ESCs, focusing on the apparent germline state enriched in 2i. We employed a GFP reporter for the key PGC specifier *Blimp1*, which faithfully reports on endogenous BLIMP1 expression in ESCs (Figure 4A). While ESCs in all four naive conditions express the undifferentiated marker CD31, ESCs in 2i contained a greater proportion of *Blimp1*-GFP$^+$ (Figure 4B).

To determine the identity of this population we isolated *Blimp1*-high, -low, or -negative ESCs, and found that all fractions express equivalent levels of the pluripotency genes, *Oct4, Sox2*, and *Nanog*, by qRT-PCR. Strikingly however, there was a highly significant upregulation of PGC markers *Nanos3, Ap2γ, Blimp1*, and *Stella*, specifically in the *Blimp1*$^+$ ESCs (Figure 4C). This was paralleled by strong repression of *Uhrf1* and *Klf4,* which are coordinately silenced uniquely in the PGC lineage. In contrast, expression of primitive endoderm genes *Gata4, Sox7*, and *Hex*, which are also associated with BLIMP1 (Ohinata et al.,

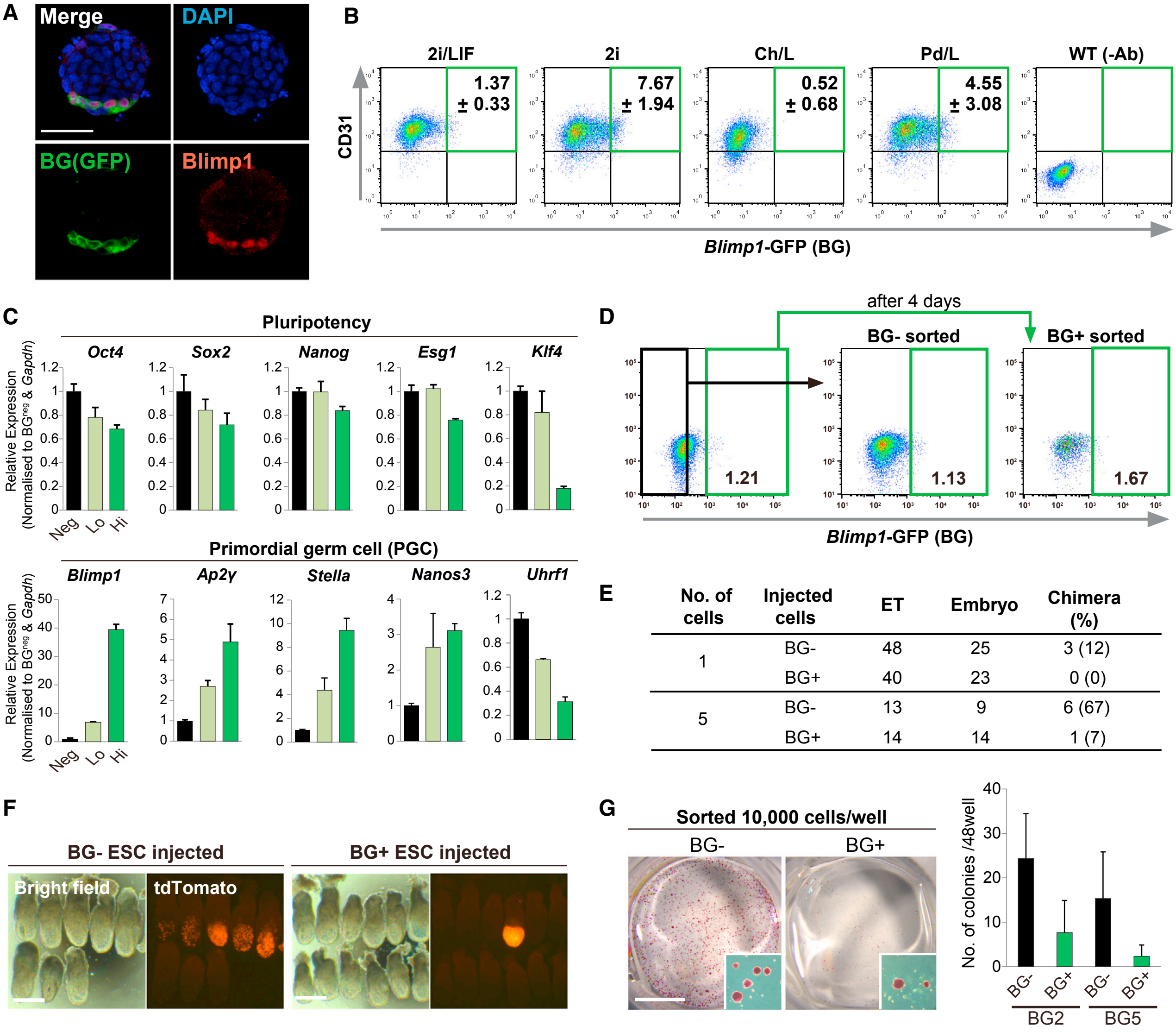

No. of cells	Injected cells	ET	Embryo	Chimera (%)
1	BG-	48	25	3 (12)
	BG+	40	23	0 (0)
5	BG-	13	9	6 (67)
	BG+	14	14	1 (7)

Figure 4. ESCs Enter a *Blimp1*-Positive PGC-like State

(A) Immunofluorescence staining of ESCs showing co-activation of *Blimp1*-GFP and endogenous BLIMP1. Scale bar, 50 μm.

(B) FACS plots of ESCs in indicated conditions showing 2i most strongly activates *Blimp1* expression.

(C) Gene expression of naive pluripotency markers (upper) and PGC markers in sorted *Blimp1*$^{-}$, *Blimp1*^{low}, and *Blimp1*^{high} ESC fractions by qRT-PCR. n = 3 values obtained from technical replicates; error bars, SEM.

(D) FACS showing that purified *Blimp1*$^{+}$ and *Blimp1*$^{-}$ ESC fractions reacquire the initial equilibrium of *Blimp1*-GFP activity in the population after 4 days.

(E) Capacity of single or pools of five *Blimp1*$^{+}$ or *Blimp1*$^{-}$ ESCs maintained in 2i/L to contribute to embryonic chimeras. ET, embryo transfers.

(F) Representative images of chimera contribution at E6.5 of constitutive H2B-tdTomato ESCs from *Blimp1*$^{+}$ or *Blimp1*$^{-}$ fractions. Scale bars, 200 μm.

(G) Alkaline phosphatase-positive colony formation by sorted *Blimp1*$^{+}$ and *Blimp1*$^{-}$ ESCs. Quantification of independent ESC lines in the right panel. Scale bar, 5 mm.

2008), were not altered. These data are consistent with *Blimp1*$^{+}$ ESCs acquiring a PGC-like transcriptional conformation (Figure S5E). We next considered that *Blimp1*$^{+}$ ESCs could either reversibly transit between positive and negative status in culture, or represent a static population. To determine this we fluorescence-activated cell sorted and re-plated *Blimp1* -positive and -negative cells. After 4 days both isolated sub-populations reacquired equivalent levels

of *Blimp1*$^+$ cells, indicating that at least some ESCs can enter and exit *Blimp1*$^+$ status at a frequency that rapidly leads to population equilibrium (Figure 4D).

To characterize *Blimp1*$^+$ ESCs functionally, we inserted a constitutive H2B-tdTomato cassette into the *Blimp1*-GFP ESC line. We then introduced either 1-cell or 5-cells from 2i/L culture into blastocysts to examine the capacity for contribution to chimeras. Whereas *Blimp1*$^-$ ESCs robustly integrated into chimeras (12% for 1-cell and 67% of embryos for 5-cell injections), *Blimp1*$^+$ ESCs contributed poorly (0% for 1-cell and 7% of embryos for 5-cell injections), despite both populations expressing high levels of naive pluripotency genes (Figures 4E and 4F). We next tested colony-formation capacity and found that *Blimp1*$^+$ ESCs generated significantly fewer alkaline-positive colonies after re-plating than *Blimp1*$^-$ ESCs (Figure 4G). These results are consistent with observations that PGCs cannot contribute to chimeras or directly form colonies, despite expressing naive pluripotency genes (Leitch et al., 2014). The combined data support the conclusion that ESCs transiently acquire a PGC-like status, with the frequency elevated in 2i-only conditions.

Klf4 Regulates a Germline Configuration in ESCs

To understand how extrinsic signals affect dynamic ESC sub-populations we sought to investigate the regulatory principles that modulate the PGC-like ESC status. The observation that *Blimp1*$^+$ are most prevalent in the 2i without LIF implies that LIF may repress entry into the germline program. To test this we found that inhibition of LIF targets JAK, but not phosphatidylinositol 3-kinase, restored the fraction of *Blimp1*-GFP$^+$ cells in 2i/L to 2i levels, suggesting that JAK/STAT3 is the critical germline-repressive pathway downstream of LIF (Figure 5A). JAK/STAT3 is known to activate multiple direct targets in ESCs, including *Klf4, Gbx2, Tfcp2l1*, and *Klf2*. Forced expression of these factors using ESCs maintained in 2i revealed *Klf4* most strongly downregulates *Blimp1* activation to levels comparable with 2i/L (Figure 5B). Subsequent immunostaining revealed that KLF4 expression within ESC colonies is inversely correlated with BLIMP1, supporting a direct relationship (Figure 5C).

To investigate this possibility we generated *Klf4*$^{-/-}$ ESCs carrying *Blimp1*-GFP using CRISPR targeting. Mutant ESCs formed colonies, proliferated normally, and maintained strong expression of NANOG (Figure 5D). Moreover, qRT-PCR profiling showed that expression of naive markers was unaltered between wild-type (WT) and *Klf4*$^{-/-}$ ESCs, with the exception of modest upregulation of *Klf2* (Figure 5E). Importantly, in the absence of *Klf4,* activation of *Blimp1*-GFP in 2i/L increased markedly to levels similar to 2i, suggesting that KLF4 is the critical mediator sufficient to drive LIF-dependent repression of the PGC program in ESCs (Figure 5F). To examine whether re-introduction of *Klf4* rescued this, we made use of a destabilized *Klf4* construct whereby exogenous KLF4 is stabilized by addition of *Shield1,* enabling tuning of protein levels. In the absence of *Shield1*, the levels of exogenous KLF4 reached levels comparable with WT, while addition of *Shield1* led to a significant overexpression (Figure 5G). We found that expressing *Klf4* at WT levels in knockout ESCs re-imposed repression of the *Blimp1*$^+$ PGC-like state in 2i/L. Moreover, elevating KLF4 levels further by addition of *Shield1* further reduced the fraction of *Blimp1*$^+$ ESCs to <0.5% (Figure 5H), with KLF4 chromatin immunoprecipitation-qPCR of the *Blimp1* promoter suggesting that this may be an indirect effect (Figure S5G). Thus, *Klf4* acts downstream of LIF/STAT3 signaling in ESCs to block dynamic entry into a germline-associated configuration.

DISCUSSION

Our study reports a spectrum of distinct transcriptional states of pluripotency that appear to order from most naive associated to most developmentally advanced. The spectrum reflects a response to culture conditions, which influence the overall ESC transcriptome and epigenome, as well as controlling access to sub-populations. The continuum of ESC states is coupled with a gradient of increasing global DNA methylation levels, with the more hypomethylated states most closely linked with a naive signature. Moreover, alternate pluripotent conformations are linked with activation of at least some distinct classes of TEs. This, in turn, appears to influence the transcriptome, potentially through a number of routes, such as opening local chromatin structure, acting as enhancers, or forming chimeric transcripts (Chuong et al., 2016). We observed that the latter mechanism is prevalent, with chimeric transcript expression often being restricted to a unique or subsets of pluripotent conditions.

Stage-specific activation of TEs has been observed during early embryonic development, and is thought to have been co-opted to drive gene regulatory networks in a stepwise manner (Thompson et al., 2016). In this way, sequential TE activation may coordinately regulate groups of genes required for successive stages of ontogeny. An example of this can be seen at the 2C embryonic stage, where multiple key genes are activated as chimeric transcripts from MT2 and MERVL elements (Macfarlan et al., 2012). The observation that the distinct ESC conformations observed here are also linked with differential TE activity suggests that TEs may contribute to overall pluripotent status. Notably it is possible that the gradient of epigenomic states across conditions partially underpins differential TE expression, as TEs are generally more prominently activated in hypomethylated

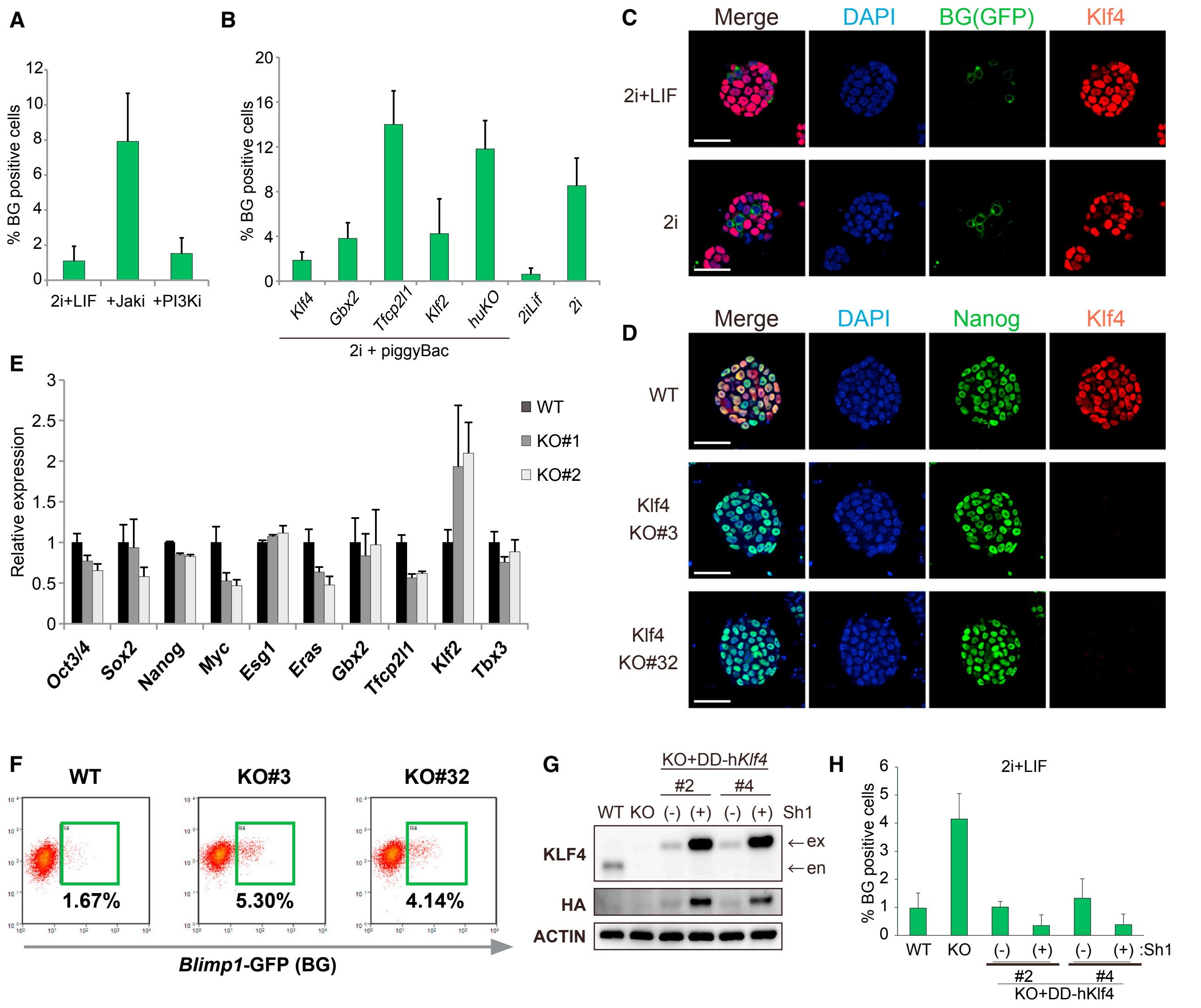

Figure 5. *Klf4* Represses the *Blimp1*-Positive State in ESCs Downstream of STAT3

(A) Percentage of *Blimp1*$^+$ ESCs in 2i/L with or without the JAK kinase inhibitor or the phosphatidylinositol 3-kinase (PI3K) inhibitor.

(B) Percentage of *Blimp1*$^+$ ESCs in 2i with forced expression of the indicated LIF target gene (A and B). n = 3 biological replicates each assayed in technical triplicate; error bars, SEM.

(C) Immunofluorescence staining of *Blimp1*-GFP and KLF4 in ESC colonies showing mutually exclusive expression. Scale bar, 50 μm.

(D) Immunofluorescence showing knockout (KO) of *Klf4* in independent ESC lines and undetectable effects on pluripotency gene NANOG. Scale bars, 50 μm.

(E) qRT-PCR analysis of gene expression in *Klf4* KO ESCs. n = 3 biological replicates each assayed in technical triplicate.

(F) FACS analysis showing a significant increase in the *Blimp1*$^+$ fraction in *Klf4* KO ESCs.

(G) Western blot showing KLF4 transgene rescue to WT-equivalent levels (−) or overexpression (+) in *Klf4* KO cells.

(H) Re-expressing KLF4 re-imposes repression of the *Blimp1*$^+$ ESC state in a dose-dependent manner. n = 3 biological replicates each assayed in technical triplicate; error bars, SEM.

ESCs here, and transition between pluripotent/epigenetic states is also linked with distinct TE responses (Walter et al., 2016). Moreover, repeat elements activated in the more derestricted epigenetic states along the spectrum, such as LTR45 and IAP, correlate well with TEs activated upon deletion of epigenetic regulators (Reichmann et al., 2012). This collectively implies a direct relationship between the epigenome, TE activity, and overall cell state.

Whether alternate in vitro pluripotent states have any functional significance is an important question. Indeed,

despite inducing broad transcriptional/epigenetic differences, the assayed culture conditions are still all capable of supporting naive pluripotency, implying that activation of the core pluripotency network overcomes wider gene expression variance. Nevertheless, the initial naive condition may subtly influence subsequent differentiation efficiency. For example, ESC propagation in Pd/L led to a poor capacity to generate the germline in vitro, but by contrast was highly efficient for the induction of nascent neuroectoderm. Likewise, culture in 2i without LIF supported high PGCLC induction, whereas Ch/L impaired such specification. It may, therefore, be important to consider the initial ESC culture condition when optimizing directed differentiation approaches, as it can leave a legacy that influences subsequent cell fate efficiency. This would imply that there is no optimal pluripotent condition to induce every cell fate with maximal efficiency, but rather a certain degree of pre-existing "bias" might be exploited according to the required endpoint. Indeed, this has recently been demonstrated by utilizing naive-like hESCs that modestly co-express primitive streak/mesodermal regulators to directly generate human PGCLCs with high efficiency (Irie et al., 2015). A further issue to consider is the apparent atrophy of imprints after extended 2i/L culture, which would be predicted to impact the developmental potential of ESCs.

A key contributor to the distinct transcriptomes between culture conditions is differential heterogeneity, as revealed at the protein level. This extends to master lineage regulators such as SOX17, GATA4, PAX6, OTX2, SOX1, and BLIMP1. Using *Blimp1*$^{+}$ cells as a paradigm, we observed that this heterogeneity is transient, and marks a sub-population of ESCs in naive conditions with PGC-like properties. This includes strong upregulation of important germline markers *Stella, Nanos3, Ap2y*, and *Blimp1*, downregulation of *Uhrf1* and *Klf4,* and poor performance in pluripotency assays despite strong expression of core pluripotency genes, which are all consistent with PGC properties. We show that activation of the PGC-like state in ESCs is directly repressed by LIF/STAT3 signaling. The critical LIF/STAT3 target is *Klf4*, which unlike other naive pluripotency genes, is repressed in PGCs in vivo (Nagamatsu et al., 2013), presumably to enable activation of the incipient germline program. Activation of *Blimp1* in ESCs may be a consequence of the negative feedback loop that operates on LIF/STAT3 signaling (Starr et al., 1997), which would promote windows of *Klf4* downregulation and transient activation of a PGC-like program. The prevalence of such cell-state dynamics in ESC populations may leave an enduring molecular memory, perhaps by enhancer priming, which influences subsequent cell responses to inductive cues. Alternatively, differences between culture conditions may reflect other parameters, such as differential metabolic profiles or chromatin states. The extent to which these possibilities are responsible for defining ESC states and responses merits further investigation.

EXPERIMENTAL PROCEDURES

All husbandry and experiments involving mice were authorized by a UK Home Office Project License 80/2637 and carried out in a Home Office-designated facility.

ESCs were maintained on gelatin-coated dishes using culture medium as specified in Figure 1A, for at least five passages prior to experimental analysis. ESCs were maintained in a humidified 37°C chamber supplemented with 5% CO_2 and passaged every 2–3 days with TrpLE. RNA-seq was performed on independent replicate samples using the TruSeq RNA Library Preparation v. 2.0 Kit (Illumina). Whole-genome bisulfite sequencing was carried out using the EZ DNA Methylation Gold Kit (Zymo Research) and Ovation Ultralow Methyl-seq Kit (NuGEN). Libraries were sequenced on a HiSeq 1500 or 2500. Differentiation assays were performed as described previously (Ying et al., 2003; Hayashi et al., 2011). CRISPR-mediated knockout of *Klf4* was achieved using dual gRNAs and the Cas9 nickase to target a critical portion of exon 3. A complete description of all methods and bioinformatics analysis is listed in the Supplemental Experimental Procedures.

ACCESSION NUMBERS

All data have been submitted to GEO under accession GEO: GSE98517.

SUPPLEMENTAL INFORMATION

Supplemental Information includes Supplemental Experimental Procedures and five figures and can be found with this article online at http://dx.doi.org/10.1016/j.stemcr.2017.05.014.

AUTHOR CONTRIBUTIONS

J.A.H. designed the study, performed experiments and bioinformatics, and wrote the manuscript. T.K. designed the study and performed experiments. S.D. performed bioinformatics analysis. M.A.S. designed and supervised the study.

ACKNOWLEDGMENTS

We thank members of the Surani lab and Dr Harry Leitch for critical reading of the manuscript and helpful discussions on the project. The *Sox1*-GFP ESCs were a kind gift from Prof. Austin Smith. The 2C::tdTomato construct was a kind gift from Dr Todd Macfarlan. Funding for this study came from a Wellcome Trust program grant (to M.A.S.), and Cancer Research UK (C6946/A14492)/Wellcome Trust (092096) core grants.

Received: April 6, 2017
Revised: May 11, 2017
Accepted: May 12, 2017
Published: June 6, 2017

REFERENCES

Blaschke, K., Ebata, K.T., Karimi, M.M., Zepeda-Martinez, J.A., Goyal, P., Mahapatra, S., Tam, A., Laird, D.J., et al. (2013). Vitamin C induces Tet-dependent DNA demethylation and a blastocyst-like state in ES cells. Nature *500*, 222–226.

Boroviak, T., Loos, R., Lombard, P., Okahara, J., Behr, R., Sasaki, E., Nichols, J., Smith, A., et al. (2015). Lineage-specific profiling delineates the emergence and progression of naive pluripotency in mammalian embryogenesis. Dev. Cell *35*, 366–382.

Bourque, G., Leong, B., Vega, V.B., Chen, X., Lee, Y.L., Srinivasan, K.G., Chew, J.L., Ruan, Y., et al. (2008). Evolution of the mammalian transcription factor binding repertoire via transposable elements. Genome Res. *18*, 1752–1762.

Cahan, P., and Daley, G.Q. (2013). Origins and implications of pluripotent stem cell variability and heterogeneity. Nat. Rev. Mol. Cell Biol. *14*, 357–368.

Chuong, E.B., Elde, N.C., and Feschotte, C. (2016). Regulatory activities of transposable elements: from conflicts to benefits. Nat. Rev. Genet. *18*, 71–86.

D'Hulst, C., Parvanova, I., Tomoiaga, D., Sapar, M.L., and Feinstein, P. (2013). Fast quantitative real-time PCR-based screening for common chromosomal aneuploidies in mouse embryonic stem cells. Stem Cell Reports *1*, 350–359.

De Los Angeles, A., Ferrari, F., Xi, R., Fujiwara, Y., Benvenisty, N., Deng, H., Hochedlinger, K., Jaenisch, R., et al. (2015). Hallmarks of pluripotency. Nature *525*, 469–478.

Dunn, S.J., Martello, G., Yordanov, B., Emmott, S., and Smith, A.G. (2014). Defining an essential transcription factor program for naive pluripotency. Science *344*, 1156–1160.

Eckersley-Maslin, M.A., Svensson, V., Krueger, C., Stubbs, T.M., Giehr, P., Krueger, F., Miragaia, R.J., Kyriakopoulos, C., et al. (2016). MERVL/Zscan4 network activation results in transient genome-wide DNA demethylation of mESCs. Cell Rep. *17*, 179–192.

Ficz, G., Hore, T.A., Santos, F., Lee, H.J., Dean, W., Arand, J., Krueger, F., Oxley, D., et al. (2013). FGF signaling inhibition in ESCs drives rapid genome-wide demethylation to the epigenetic ground state of pluripotency. Cell Stem Cell *13*, 351–359.

Goke, J., Lu, X., Chan, Y.S., Ng, H.H., Ly, L.H., Sachs, F., and Szczerbinska, I. (2015). Dynamic transcription of distinct classes of endogenous retroviral elements marks specific populations of early human embryonic cells. Cell Stem Cell *16*, 135–141.

Grow, E.J., Flynn, R.A., Chavez, S.L., Bayless, N.L., Wossidlo, M., Wesche, D.J., Martin, L., Ware, C.B., et al. (2015). Intrinsic retroviral reactivation in human preimplantation embryos and pluripotent cells. Nature *522*, 221–225.

Habibi, E., Brinkman, A.B., Arand, J., Kroeze, L.I., Kerstens, H.H., Matarese, F., Lepikhov, K., Gut, M., et al. (2013). Whole-genome bisulfite sequencing of two distinct interconvertible DNA methylomes of mouse embryonic stem cells. Cell Stem Cell *13*, 360–369.

Hackett, J.A., and Surani, M.A. (2014). Regulatory principles of pluripotency: from the ground state up. Cell Stem Cell *15*, 416–430.

Hackett, J.A., Dietmann, S., Murakami, K., Down, T.A., Leitch, H.G., and Surani, M.A. (2013a). Synergistic mechanisms of DNA demethylation during transition to ground-state pluripotency. Stem Cell Reports *1*, 518–531.

Hackett, J.A., Sengupta, R., Zylicz, J.J., Murakami, K., Lee, C., Down, T.A., and Surani, M.A. (2013b). Germline DNA demethylation dynamics and imprint erasure through 5-hydroxymethylcytosine. Science *339*, 448–452.

Hall, J., Guo, G., Wray, J., Eyres, I., Nichols, J., Grotewold, L., Morfopoulou, S., Humphreys, P., et al. (2009). Oct4 and LIF/Stat3 additively induce Kruppel factors to sustain embryonic stem cell self-renewal. Cell Stem Cell *5*, 597–609.

Hayashi, K., Ohta, H., Kurimoto, K., Aramaki, S., and Saitou, M. (2011). Reconstitution of the mouse germ cell specification pathway in culture by pluripotent stem cells. Cell *146*, 519–532.

Huang, K., Maruyama, T., and Fan, G. (2014). The naive state of human pluripotent stem cells: a synthesis of stem cell and preimplantation embryo transcriptome analyses. Cell Stem Cell *15*, 410–415.

Irie, N., Weinberger, L., Tang, W.W., Kobayashi, T., Viukov, S., Manor, Y.S., Dietmann, S., Hanna, J.H., et al. (2015). SOX17 is a critical specifier of human primordial germ cell fate. Cell *160*, 253–268.

Klein, A.M., Mazutis, L., Akartuna, I., Tallapragada, N., Veres, A., Li, V., Peshkin, L., Weitz, D.A., et al. (2015). Droplet barcoding for single-cell transcriptomics applied to embryonic stem cells. Cell *161*, 1187–1201.

Kolodziejczyk, A.A., Kim, J.K., Tsang, J.C., Ilicic, T., Henriksson, J., Natarajan, K.N., Tuck, A.C., Gao, X., et al. (2015). Single cell RNA-sequencing of pluripotent states unlocks modular transcriptional variation. Cell Stem Cell *17*, 471–485.

Leitch, H.G., Mcewen, K.R., Turp, A., Encheva, V., Carroll, T., Grabole, N., Mansfield, W., Nashun, B., et al. (2013). Naive pluripotency is associated with global DNA hypomethylation. Nat. Struct. Mol. Biol. *20*, 311–316.

Leitch, H.G., Okamura, D., Durcova-Hills, G., Stewart, C.L., Gardner, R.L., Matsui, Y., and Papaioannou, V.E. (2014). On the fate of primordial germ cells injected into early mouse embryos. Dev. Biol. *385*, 155–159.

Macfarlan, T.S., Gifford, W.D., Driscoll, S., Lettieri, K., Rowe, H.M., Bonanomi, D., Firth, A., Singer, O., et al. (2012). Embryonic stem cell potency fluctuates with endogenous retrovirus activity. Nature *487*, 57–63.

Marks, H., Kalkan, T., Menafra, R., Denissov, S., Jones, K., Hofemeister, H., Nichols, J., Kranz, A., et al. (2012). The transcriptional and epigenomic foundations of ground state pluripotency. Cell *149*, 590–604.

Morgani, S.M., Canham, M.A., Nichols, J., Sharov, A.A., Migueles, R.P., Ko, M.S., and Brickman, J.M. (2013). Totipotent embryonic stem cells arise in ground-state culture conditions. Cell Rep. *3*, 1945–1957.

Nagamatsu, G., Kosaka, T., Saito, S., Honda, H., Takubo, K., Kinoshita, T., Akiyama, H., Sudo, T., et al. (2013). Induction of pluripotent stem cells from primordial germ cells by single reprogramming factors. Stem Cells *31*, 479–487.

Nichols, J., and Smith, A. (2009). Naive and primed pluripotent states. Cell Stem Cell *4*, 487–492.

Ohinata, Y., Sano, M., Shigeta, M., Yamanaka, K., and Saitou, M. (2008). A comprehensive, non-invasive visualization of primordial germ cell development in mice by the Prdm1-mVenus and Dppa3-ECFP double transgenic reporter. Reproduction *136*, 503–514.

Pauklin, S., and Vallier, L. (2013). The cell-cycle state of stem cells determines cell fate propensity. Cell *155*, 135–147.

Peaston, A.E., Evsikov, A.V., Graber, J.H., de Vries, W.N., Holbrook, A.E., Solter, D., and Knowles, B.B. (2004). Retrotransposons regulate host genes in mouse oocytes and preimplantation embryos. Dev. Cell *7*, 597–606.

Piccolo, F.M., Bagci, H., Brown, K.E., Landeira, D., Soza-Ried, J., Feytout, A., Mooijman, D., Hajkova, P., et al. (2013). Different roles for Tet1 and Tet2 proteins in reprogramming-mediated erasure of imprints induced by EGC fusion. Mol. Cell *49*, 1023–1033.

Reichmann, J., Crichton, J.H., Madej, M.J., Taggart, M., Gautier, P., Garcia-Perez, J.L., Meehan, R.R., and Adams, I.R. (2012). Microarray analysis of LTR retrotransposon silencing identifies Hdac1 as a regulator of retrotransposon expression in mouse embryonic stem cells. PLoS Comput. Biol. *8*, e1002486.

Sheik Mohamed, J., Gaughwin, P.M., Lim, B., Robson, P., and Lipovich, L. (2010). Conserved long noncoding RNAs transcriptionally regulated by Oct4 and Nanog modulate pluripotency in mouse embryonic stem cells. RNA *16*, 324–337.

Starr, R., Willson, T.A., Viney, E.M., Murray, L.J., Rayner, J.R., Jenkins, B.J., Gonda, T.J., Alexander, W.S., et al. (1997). A family of cytokine-inducible inhibitors of signalling. Nature *387*, 917–921.

Thompson, P.J., Macfarlan, T.S., and Lorincz, M.C. (2016). Long terminal repeats: from parasitic elements to building blocks of the transcriptional regulatory repertoire. Mol. Cell *62*, 766–776.

Tonge, P.D., Corso, A.J., Monetti, C., Hussein, S.M., Puri, M.C., Michael, I.P., Li, M., Lee, D.S., et al. (2014). Divergent reprogramming routes lead to alternative stem-cell states. Nature *516*, 192–197.

Torres-Padilla, M.E., and Chambers, I. (2014). Transcription factor heterogeneity in pluripotent stem cells: a stochastic advantage. Development *141*, 2173–2181.

von Meyenn, F., Iurlaro, M., Habibi, E., Liu, N.Q., Salehzadeh-Yazdi, A., Santos, F., Petrini, E., Milagre, I., et al. (2016). Impairment of DNA methylation maintenance is the main cause of global demethylation in naive embryonic stem cells. Mol. Cell *62*, 983.

Walter, M., Teissandier, A., Perez-Palacios, R., and Bourc'his, D. (2016). An epigenetic switch ensures transposon repression upon dynamic loss of DNA methylation in embryonic stem cells. Elife *5*, e11418.

Wan, M., Liang, J., Xiong, Y., Shi, F., Zhang, Y., Lu, W., He, Q., Yang, D., Chen, R., Liu, D., et al. (2012). The trithorax group protein Ash2l is essential for pluripotency and maintaining open chromatin in embryonic stem cells. J Biol. Chem. *288*, 5039–5048.

Wang, J., Xie, G., Singh, M., Ghanbarian, A.T., Rasko, T., Szvetnik, A., Cai, H., Besser, D., et al. (2014). Primate-specific endogenous retrovirus-driven transcription defines naive-like stem cells. Nature *516*, 405–409.

Weinberger, L., Ayyash, M., Novershtern, N., and Hanna, J.H. (2016). Dynamic stem cell states: naive to primed pluripotency in rodents and humans. Nat. Rev. Mol. Cell Biol. *17*, 155–169.

Wray, J., Kalkan, T., and Smith, A.G. (2010). The ground state of pluripotency. Biochem. Soc. Trans. *38*, 1027–1032.

Wu, J., and Izpisua Belmonte, J.C. (2015). Dynamic pluripotent stem cell states and their applications. Cell Stem Cell *17*, 509–525.

Wu, J., Okamura, D., Li, M., Suzuki, K., Luo, C., Ma, L., He, Y., Li, Z., et al. (2015). An alternative pluripotent state confers interspecies chimaeric competency. Nature *521*, 316–321.

Yamaguchi, S., Shen, L., Liu, Y., Sendler, D., and Zhang, Y. (2013). Role of Tet1 in erasure of genomic imprinting. Nature *504*, 460–464.

Ye, S., Li, P., Tong, C., and Ying, Q.L. (2013). Embryonic stem cell self-renewal pathways converge on the transcription factor Tfcp2l1. EMBO J. *32*, 2548–2560.

Ying, Q.L., Stavridis, M., Griffiths, D., Li, M., and Smith, A. (2003). Conversion of embryonic stem cells into neuroectodermal precursors in adherent monoculture. Nat. Biotechnol. *21*, 183–186.

Ying, Q.L., Wray, J., Nichols, J., Batlle-Morera, L., Doble, B., Woodgett, J., Cohen, P., and Smith, A. (2008). The ground state of embryonic stem cell self-renewal. Nature *453*, 519–523.

Zalzman, M., Falco, G., Sharova, L.V., Nishiyama, A., Thomas, M., Lee, S.L., Stagg, C.A., Hoang, H.G., Yang, H.T., Indig, F.E., et al. (2010). Zscan4 regulates telomere elongation and genomic stability in ES cells. Nature *464*, 858–863.

Stem Cell Reports, Volume *8*

Supplemental Information

Activation of Lineage Regulators and Transposable Elements across a Pluripotent Spectrum

Jamie A. Hackett, Toshihiro Kobayashi, Sabine Dietmann, and M. Azim Surani

EXTENDED EXPERIMENTAL PROCEDURES

Culture of ESCs

Undifferentiated mouse ESCs from 129 (129X1/SvJ) ('CES5'), B6 (C57BL6/J) ('BK2'), *Stella*-GFP/2C::tdTomato, *Blimp1*-GFP, ΔPE-*Oct4*-GFP and *Sox1*-GFP (transgenic derived) strains were maintained on gelatin-coated dishes in the specified culture mediums, for at least 5 passages (see Fig 1A). ESC were propagated in a humidified 37°C chamber supplemented with 5% CO_2. ESC culture media was replaced with fresh media every day and cells were routinely passaged every 2-3 days. For basal medium, N2B27 medium was prepared according to a published protocol or purchased as NDIF 227 from Takara bio (Ying et al., 2008). For GMEM or KO-DMEM based media, each medium purchased from Thermo scientific was supplemented with 0.1 mM 2-mercaptoethanol (Thermo scientific), 0.1 mM non-essential amino acids (Thermo scientific), 1 mM sodium pyruvate (Thermo scientific), 1% L-glutamine (Sigma) and penicillin/streptomycin (Thermo scientific). For signalling or supplementation, 10% fetal bovine serum (Thermo scientific), 10% KSR (Thermo scientific), 1 μM MEK inhibitor PD0325901 (MACS), 3 μM GSK3 inhibitor CHIR99021 (MACS), and 1,000 U/ml of mouse LIF (Cambridge Stem Cell Institute) are added respectively according to the condition.

Embryo Culture and Manipulation

Preparation of C57BL6/J x BCF1 intercrossed embryos was carried out according to standard protocols. In brief, 8-cell/morula stage embryos were collected in M2 medium (sigma) from the oviduct and uterus of mice 2.5 days *post coitum* (dpc). For micro-manipulation, sorted *Blimp1*-GFP ESCs were suspended in ESC culture medium. A piezo-driven micro-manipulator (Prime Tech, Tokyo, Japan) was used to drill zona pellucida under the microscope and 1 or 5 ESCs were introduced into the space between zona pellucida and blastomeres of 8-cell/morula stage embryos. After injection, embryos were cultured until the next day in KSOM medium (Millipore). Morula or early blastocysts were subsequently transferred into the uteri of pseudopregnant recipient MF1 female mice (2.5 dpc).

ESC Differentiation Protocols

Specification of primordial germ cell-like cells (PGCLC) was performed as previously described, with minor modifications (Hayashi et al., 2011). ESC were maintained in the indicated culture medium on gelatin coated plates. Induction into epiblast-like cells (EpiLC) was performed by passaging $1.2x10^5$ ESC onto fibronectin coated 12 well plates containing N2B27 supplemented with 12.5ng/ml FGF2, 20ng/ml Activin-A (both Cambridge Stem Cell Institute) and 1% KSR for 40 hours. PGCLCs were subsequently specified in embryoid bodies formed in ultra-low attachment 96-well plates (Corning) containing GK15 supplemented with 500ng/ml BMP4, 500ng/ml BMP8, 50ng/ml EGF, 100ng/ml SCF (all R&D Systems) and 1,000 U/ml mouse LIF. Induction of monolayer neuro-ectoderm differentiation was performed as previously described (Ying et al., 2003). Briefly, *Sox1*-GFP ESC maintained in the indicated ESC culture medium were washed with PBS thrice, and passaged to gelatin-coated 6-well plates containing NDIF 227 (Takara) without supplements at optimal density.

Analysis of *Blimp1*-GFP ESC

To analyse the percentage of *Blimp1*-GFP subpopulation by FACS, cells were trypsinized and suspended in PBS containing 3% FCS. To check CD31 expression, cells were stained with APC-conjugated anti-CD31 antibody (eBioscience) for 30-60 min on ice. Cells were analyzed by FACS LSRFrotessa (BD Bioscience) or sorted by Moflo (Beckman Coulter). All data were

re-analyzed by Flowjo software. For bulk colony formation assay, 10,000 cells were sorted and seeded on a gelatin coated well of 24 well plate. After 5 days, cells were fixed and stained with Alkaline Phosphatase Detection Kit (Sigma) according to manufacturer's protocol. For single cell colony formation assay, single Blimp1-GFP positive or negative cells were sorted into ESC medium on gelatin coated well of 96 well plate. After 5 days, numbers of well has undifferentiated ESC colony were counted. For signaling inhibition experiments, 10 uM Jak inhibitor I (sigma); 5 uM LY294002 (Sigma), were added to N2B27+2i/L medium and cells analyzed by FACS after 2-3 days culture. For screening TFs to suppress *Blimp1*-GFP positive population, a PiggyBac vector containing *CAG* promoter, genes shown in Fig 5B and *IRES-Puro* cassette were co-transfected with a PBase expression vector into *Blimp1*-GFP ESCs cultured in 2i using lipofectamine 2000 (Thermo scientific). Two days after transfection, 1.0ug/ml puromycin was added to medium to select cells that stably express the transgene. Cells were analysed 7-10 days after transfection.

Generation and Rescue of *Klf4* KO ESCs

For *Klf4* KO ESCs, the CRISPR/Cas9 double nickase system was used. Two guide RNAs coding (5'-GGCAGGGCCGCTGCTCGCCG-3' and 5'- CAGCTATCCGATCCGGGCCG-3') were inserted into *BsgI* restriction enzyme site of pX335 vector. These plasmids were co-tranfected with vector containing Puromycin resistance gene into *Blimp1*-GFP ESCs using lipofectamine 2000. Two days after transfection, 1.0ug/ml puromycin was added to the medium and drug resistant (transfected) cells were selected for a further 2 days. After the selection, ESC colonies were picked up and screened for homozygous KO by PCR, Western blot (data not shown), and IF. To rescue *Klf4* KO ESCs, a variant of Shield1/destabilized domain (DD) (Clontech) inducible expression system was used. A PiggyBac vector containing *CAG* promoter, DD fused HA tagged *Klf4* and *IRES-Puro* cassette were co-transfected with PBase expression vector into *Klf4* KO ESCs using lipofectamine 2000 (Thermo scientific). After puromycin selection, the resistant colonies were picked and the expression level of *Klf4* was validated by Western blot.

Generation of *Stella*-GFP & 2C::tdTomato double-reporter ESCs

For construction of the targeting vector for the *Rosa26* locus, a splice accepter sequence, Neomycin resistance gene with pA and insulator sequence, *2C-tdTomato* cassette amplified from 2C::tdTomato Reporter (a gift from Prof. Samuel Pfaff), and *IRES-Puro* were inserted into *XbaI* site of pROSA26-SwaI with the InFusion cloning kit (Takara Bio). For gene targeting, electroporation was carried out using *Stella*-EGFP transgenic (shorter SH6 transgene construct) ESCs. Briefly 4 x 10^6 ESC suspended in PBS were mixed with 20 μg linearized targeting vector, and then, were transferred to a Gene Pulser cuvette (Bio-Rad, Richmond, CA). Electroporation was carried out at 230 V, 250 μF in Gene Pulser equipment (Bio-Rad). After electroporation, ESCs were seeded on gelatin-coated dish, and 24 h later, 400μg/ml G418 (Sigma) was added to the culture medium. After drug selection, colonies were picked up and screened for correct targeting by PCR.

Immunofluorescence

Cultured ESCs were fixed in 4% paraformaldehyde for 10 min at room temperature. After permeabilisation with 0.25% triton-X/PBS for 10 min, ESC were blocked with 0.1% triton-X/ MAXblock™ blocking medium (Active Motif) for 30 min, and subsequently incubated with primary antibodies for 1-2 hr at RT or overnight in a cold room. Primary antibodies against

NANOG (eBioMLC-51; eBioscience), OTX2 (AB9566; abcam), PAX6 (PRB-278P; Covance), BLIMP1 (6D3; eBioscience), GATA4 (EPR4768; abcam), SOX17 (AF1924; R&D systems), KLF4 (AF3158; R&D systems), and EGFP (GF090R; Nacalai tesque) were used. Following several washes with 0.1% triton-X/PBS, cells were incubated with Alexa Fluor-conjugated secondary antibodies (Thermo Scientific) for 1 hr at RT. Following antibody treatment, cells were stained with DAPI (Sigma) to mark nuclei and were observed using a confocal laser scanning microscope.

Gene Expression & Western Blot analysis

For qRT-PCR total RNA was isolated and DNAse treated using the RNeasy mini kit (Qiagen), and reverse transcribed using Superscript III (Invitrogen). Gene expression was quantitated in quadriplucate using JumpStart SYBR green (Sigma) qPCR reagent and gene-specific primer sets on a QuantStudio 6 Flex Real-Time PCR System (Applied Biosystems), and normalised to *Gapdh* and *Arbp*. For western blots whole-cell extracts were prepared from WT or *Klf4* KO ESCs in lysis buffer composed of 50mM Tris-HCl (pH7.5), 0.15M NaCl, 0.1% SDS, 1% Triton X-100, 1% sodium deoxycholate and Complete-mini EDTA-free (Roche). After electrophoresis, proteins were transferred to nitrocellulose membranes. Membranes were incubated in blocking buffer containing skimmed milk and probed with Primary antibodies against KLF4 (goat IgG; R&D systems) and ACTIN (mouse IgG; sigma). Horseradish peroxidase-conjugated secondary antibodies against goat or mouse IgG were added (Dako). Blots were developed using SuperSignal West Pico Chemiluminescent Substrate (Thermo Scientific).

NGS Library Preparation

For RNA-seq, replicate ESC samples for each background were collected at independent timepoints (2 passages apart) following their culture in the specified media (total replicates per culture parameter n=4). 1μg of total RNA was extracted with RNeasy mini isolation kit (Qiagen), using on-column DNase steps. The poly-A fraction was isolated, fragmented and reverse transcribed into cDNA using the TruSeq RNA Library Prep kit v2.0, according to the manufacture's protocol. Double-stranded cDNA was end-repaired and Illumina adapters were ligated. Adapter-ligated DNA was purified with AMPure XP beads (Beckman Coulter) and amplified by PCR enrichment for 10 cycles. Quality control testing was carried using a TapeStation 2200 (Agilent). Individual samples were subsequently combined into multiplexed libraries and subjected to single-end 50bp sequencing on a HiSeq1500 (Illumina). For bisulfite sequencing libraries, genomic DNA was isolated with the Blood and Tissue kit (Qiagen) and sonicated to an average of 280bp in a cooled Bioruptor (Diagenode). 150ng of fragmented DNA was end-repaired and methylated indexed adapters were ligated using Ovation Ultralow Methyl-Seq Library system (Nugen). Bisulfite conversion was subsequently carried out using the EZ DNA Methylation Gold kit (Zymo Research), following the manufacture's recommendations. Lambda DNA was spiked in at 0.1% or 0.5% to track conversion efficiency. Bisulfite converted DNA was purified and amplified by PCR enrichment for 8 cycles using KAPA HiFi Uracil+ polymerase (Kapa bioscience). Individual samples were subsequently combined into multiplexed libraries and subjected to paired-end 125bp or single-end 100bp sequencing on a HiSeq1500 (Illumina). All data has been submitted to GEO under accession GSE98517.

Bioinformatics

RNA-seq analysis. Adapters were removed and reads were quality-trimmed using *TrimGalore*. Trimmed reads were aligned to the mouse reference genome (GRCm38/mm10) by using *TopHat2* (http://ccb.jhu.edu/software/tophat, version: 2.0.13) guided by ENSEMBL gene models. Raw counts per repeat regions and gene regions were obtained by *featureCounts.* Replicates were evaluated, counts were normalized and differential expression of transcripts was determined by the *R Bioconductor DESeq* package (www.bioconductor.org). Expression levels were further normalized by transcript length (per kB) where indicated. Transcript annotations in all bioinformatics analyses were based on Ensembl (Release 78). Principal component analysis was performed by singular value decomposition of the scaled expression values using the R svd() function (3D), and with the PCA function within *SeqMonk* software using log transformed merged transcript probes without length correction (2D). Gene Ontology (GO) term enrichment analyses were performed with the *Bioconductor topGO* package and the DAVID online tool v6.8 (https://david.ncifcrf.gov).

SOM analysis. All pairwise differentially expressed gene sets across the selected conditions were determined by DESeq (p value < 0.01, abs(log2FC) > 2). Self-organizing maps (SOMs) implemented by the *R kohonen()* package were trained to characterize distinct gene sets with similar transcriptional profiles in an unsupervised analysis. The size of the hexagonal SOM grid was set such that each node contained on average ~50 genes after classification.

*Repeat expression analysis***.** The mouse RepeatMasker annotation file (UCSC GRCm38/mm10) was downloaded from UCSC Tables; tRNA and rRNA repeat annotations were discarded. Trimmed reads were aligned to the mouse reference genome with *bowtie* (paramters: -m 1 –v 2 –best -strata) allowing for two mismatches and filtering uniquely mapping reads only. Raw counts per repeat regions and gene regions were obtained by *featureCounts* (parameter: -O) allowing for multiple feature assignments. Counts per repeat region were normalized by a factor between 0 and 1 obtained by the total number of counts in all genic regions divided by 10E+7. Normalized counts per retrotransposon family were pooled, and further normalized by the summed length of all individual repeat loci. Differential expression of families of retrotransposons and *p* values were estimated by the Bioconductor *DESeq* package. The fraction of active repeat loci per family was obtained by using an expression threshold of log2(normalized expression level) > 2 for each genomic repeat region.

Chimeric transcripts. Chimeric transcripts were obtained by extracting 50 nt reads that splice from repeat loci into genic exons based on the alignments generated by *TopHat2*. Repeat loci were required to be located 50 kB upstream of the transcriptional start sites of the largest annotated transcript per gene. i.e. potential splicing from intronic repeats to genic exons was not considered. A minimum of 2 independent spliced TE-gene chimeric reads was required to confirm the splicing event.

Whole genome bisulfite sequencing analysis. Bisulfite converted-reads were quality-trimmed with *Trim Galore*. Trimmed reads were then mapped to a computationally bisulfite-converted mouse reference genome (GRCm38/mm10) using *Bismark* (version: 0.7.12; parameter settings: '*-n 2 -l 40*') tolerating two non-cytosine mismatches. Potential PCR duplicates were removed using *samtools rmdup*. CpG methylation calls were extracted from deduplicated mapping output using the Bismark methylation extractor in single-end mode. CpG methylation calls

were analysed using R and *SeqMonk* software. To calculate CpG methylation levels the genome was partitioned into consecutive 5-kb tiles or probes corresponding to defined genomic features covered by at least 5 CpGs, and percentage methylation was calculated using the bisulfite feature methylation pipeline in *SeqMonk*. Respective genomic features were defined using UCSC table browser annotations, *RepeatMasker* annotations or published datasets. Heatmaps were generated using the 'heatmap.2' function within R.\

Antibodies & Oligo Sequences

Antibodies

Antigen	Company	Clone number	Cat. number	Application
CD31	Biolegend	MEC13.3	557377	FACS
NANOG	eBioscience	eBioMLC-51	14-5761-80	IF
OTX2	Millipore		AB9566	IF
PAX6	Covance		PRB-278P	IF
BLIMP1	eBioscience	6D3	14-5963-82	IF
GATA4	Abcam	EPR4768	ab134057	IF
SOX17	R&D systems		AF1924	IF
KLF4	R&D systems		AF3158	IF
KLF4	Abcam		ab129473	WB
EGFP	Nacalai tesque	GF090R	04404-26	IF
HA	CST	6E2	2367S	WB
β-Actin	CST	8H10D10	3700T	WB

qRT-PCR

Gene	Forward	Reverse
Arbp	CAAAGCTGAAGCAAAGGAAGAG	AATTAAGCAGGCTGACTTGGTTG
Oct4	CCAATCAGCTTGGGCTAGAG	CTGGGAAAGGTGTCCCTGTA
Sox2	CATGAGAGCAAGTACTGGCAAG	CCAACGATATCAACCTGCATGG
Nanog	ACCTGAGCTATAAGCAGGTTAAGAC	GTGCTGAGCCCTTCTGAATCAGAC
Esg1	AAGGAGTGCTGAAGCTGGAGG	CAGCTTAACCTGCATCCAGGTC
Klf4	CGTCCCAGTCACAGTGGTAA	AAAAGAACAGCCACCCACAC
Blimp1	AAACGTGTGGGTACGACCTT	CCTTGAAACTTCACGGAGCC
Ap2g	TGAAGATGAAGCTGGGCTTT	TCCATTCTCTTCCGGTTCAG
Stella	AGGCTCGAAGGAAATGAGTTT	TCCTAATTCTTCCCGATTTTC
Nanos3	CACTACGGCCTAGGAGCTTGG	TGATCGCTGACAAGACTGTGG
Uhrf1	CCCCTCGCAACGGAAGAGCG	CGCCCACCACACACATGGCA
Gata4	AGGCACATGACCCATCACACA	AGAGGAAGGGAAGGCACCATG
Sox7	CCACAGTCCTTTGGCTGTCC	TACACGTGTCCAAGGGCAGA
Myc	TAACTCGAGGAGGAGCTGGA	GCCAAGGTTGTGAGGTTAGC
Eras	GTAGCTGTGGCTGCTCTGTAG	GATGTCTGTGGTAACTTGGTCG
Gbx2	GTGCCCAAAGGTAAACAGGA	AAATCAACCGACTGCTCTGC
Tfcp2l1	TGGCTACCACATCATCCTGA	GCTTGTCAGGTGAGACAGCA
Klf2	ACCAAGAGCTCGCACCTAAA	GTGGCACTGAAAGGGTCTGT
Tbx3	TTATTTCCAGGTCAGGAGATG	GGTCGTTTGAACCAAGTCCCT
Sox17	TTCTGTACACTTTAATGAGGCTGTTC	TTGTGGGAAGTGGGATCAAG
Dnmt3b	CTCGCAAGGTGTGGGCTTTTGTAAC	CTGGGCATCTGTCATCTTTGCACC

ChIP

Gene	Forward	Reverse
chr10:44,458,383 -44,458,520	GGGGGAGAAAAGAAAAGTTAAAA	CGACCTTGGTAAGGAACCAG
chr10:44,458,522 -44,458,721	CTCTCATGTCCACCCAGTCC	GCGGCCGTAGAAAAGGAG
chr10:44,458,713 -44,458,912	AGTGAGCGAGCGACTGACTA	GCGGCTGGTAGGAGTGAAT
chr10:44,459,053 -44,459,152	GGGGACTCCTCCTCAAAAGA	TGCATGTGCTGCCAAAATAC
chr10:44,392,037 -44,392,236	ACCCATCTTTGTCTGGGATG	AGCCCTGGAGGAGGAACTT
Oct3/4 DE (PC)	GACGGCAGATGCATAACAAA	AGGAAGGGCTAGGACGAGAG
RLP30 (NC)	#7015P in SimpleChIP Kit	

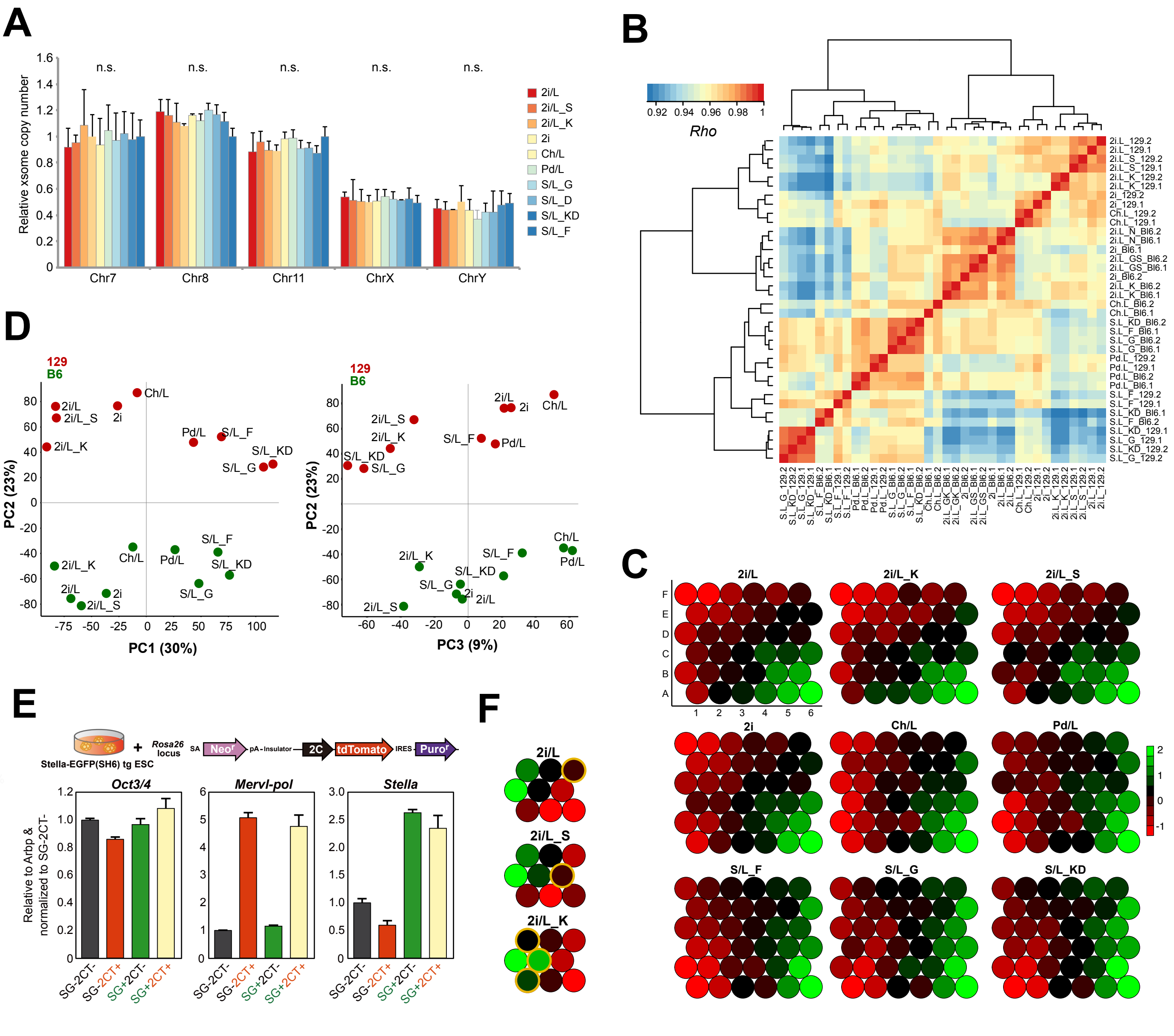

Figure S1. **Transcriptional characterisation of ESC states.** (A) Relative copy number of most commonly anueploid autosomes, and sex chromosomes, following transition to each ESC culture condition by qRT-PCR. (B) Correlation between individual replicates of RNA –seq. (C) Self-organising maps (SOM). Each circle represents the expression level of subset of all genes with similar co-expression dynamics in each ESC condition. The genes represented in position A2, for example, are highly expressed in 2i/L_K relative to other conditions. (D) Principal component analysis of ESC transcriptomes by culture condition and strain using an independent algorithm from Fig 1D (main article). (E) Expression level of indicated genes by qRT-PCR in *Stella*-GFP & 2C::tdTomato ESC, showing correlation between reporter and endogenous loci. (F) SOM comparing the addition of serum or KSR to naïve 2i/L culture conditions. Yellow circles represent genes subsets most significantly changing.

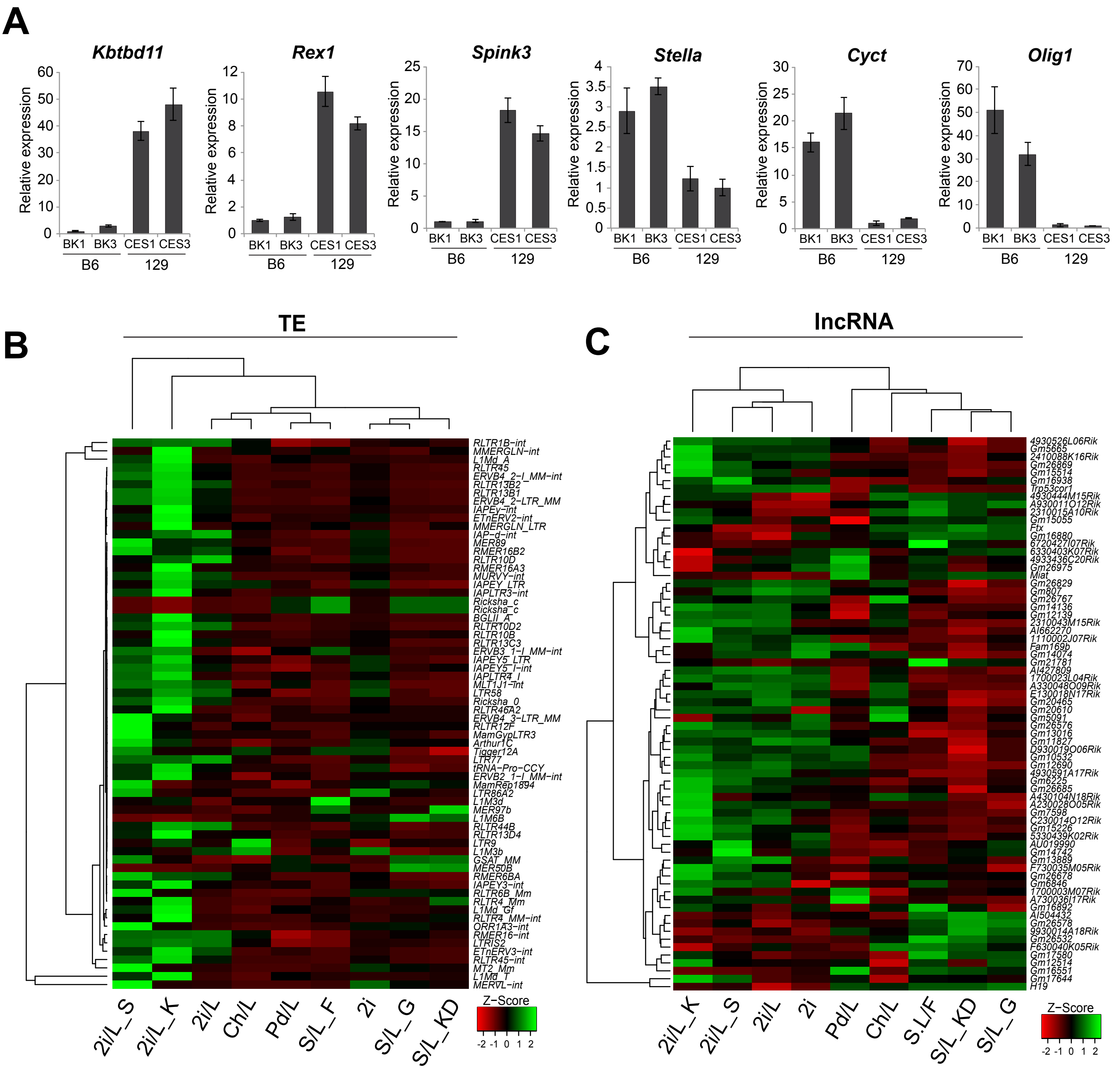

Figure S2. Expression of repetitive and non-coding elements. (A) Validation of differential expression between B6 and 129 ESC by qRT-PCR using independent lines from Fig 1. (B) Heatmap showing relative expression of transposable element (TE) classes identified as significantly differentially expressed between at least one pairwise comparison of culture conditions. (C) Heatmap showing relative expression of long noncoding RNAs (lncRNA) identified as significantly differentially expressed between at least one pairwise comparison of culture conditions. All analysis was performed on combined 129 and B6 datasets (n=4 per condition) to identify robust changes due to culture parameters.

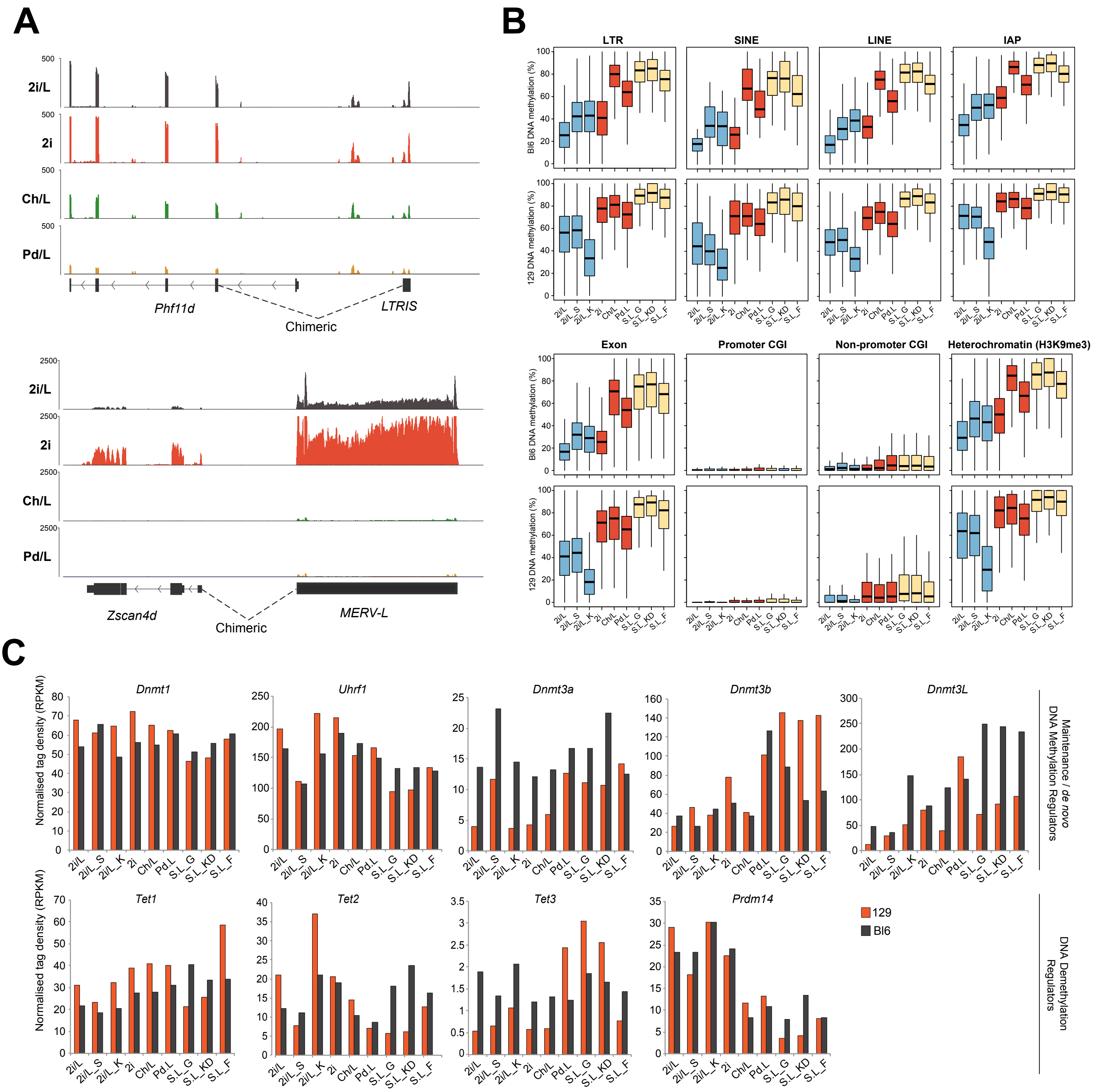

Figure S3. Chimeric and epigenomic analysis in alternative ESC states. (A) Genome view of informatically identified chimeric transcripts of *Phf11d* and *Zscan4d*, which are derived from an upstream LTR element in an ESC-state specific manner. (B) Boxplots showing median global DNA methylation levels across multiple repeat (upper) or genomic features (lower) in indicated ESC conditions and backgrounds. (C) Expresssion of multiple genes involved in DNA (de)methylation, in 129 and B6 ESC. The transcriptional levels of these genes do not definitively correlate with the global DNA methylation level across culture conditions and genetic background.

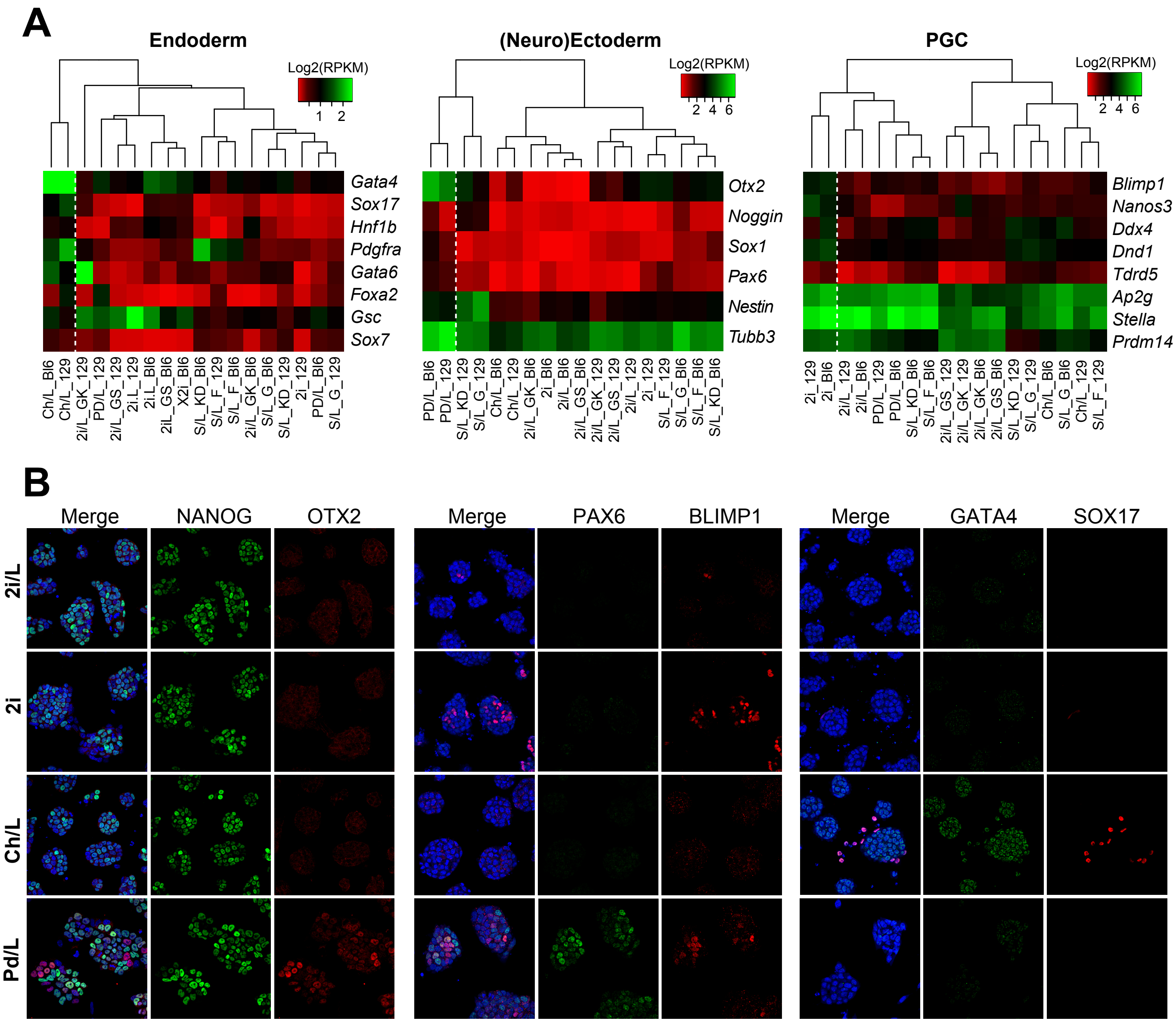

Condition	Sox17/Gata4	Pax6	Blimp1	Otx2
2i/L	0%	0%	1.6%	0%
2i	1.6%	0%	12.9%	0%
Ch/L	11.0%	0%	0%	0%
Pd/L	0%	26.7%	10.6%	37.6%

Figure S4. Activation of master lineage-regulators in naïve ESC culture conditions. (A) Heatmap showing absolute expression of key master regulators for endoderm, (neuro)ectorderm and primordial germ cells (PGC). Unbiased hierarchical clustering shows Pd/L, Ch/L and 2i, respectively, each segregate separately from all other conditions based on expression if these primary germ layer genes. (B) Immunofluorescence of master germ layer regulators confirms protein expression in only a specific culture condition, whilst remaining silenced in all others. Table shows quantification of strong-positive cells in each condition.

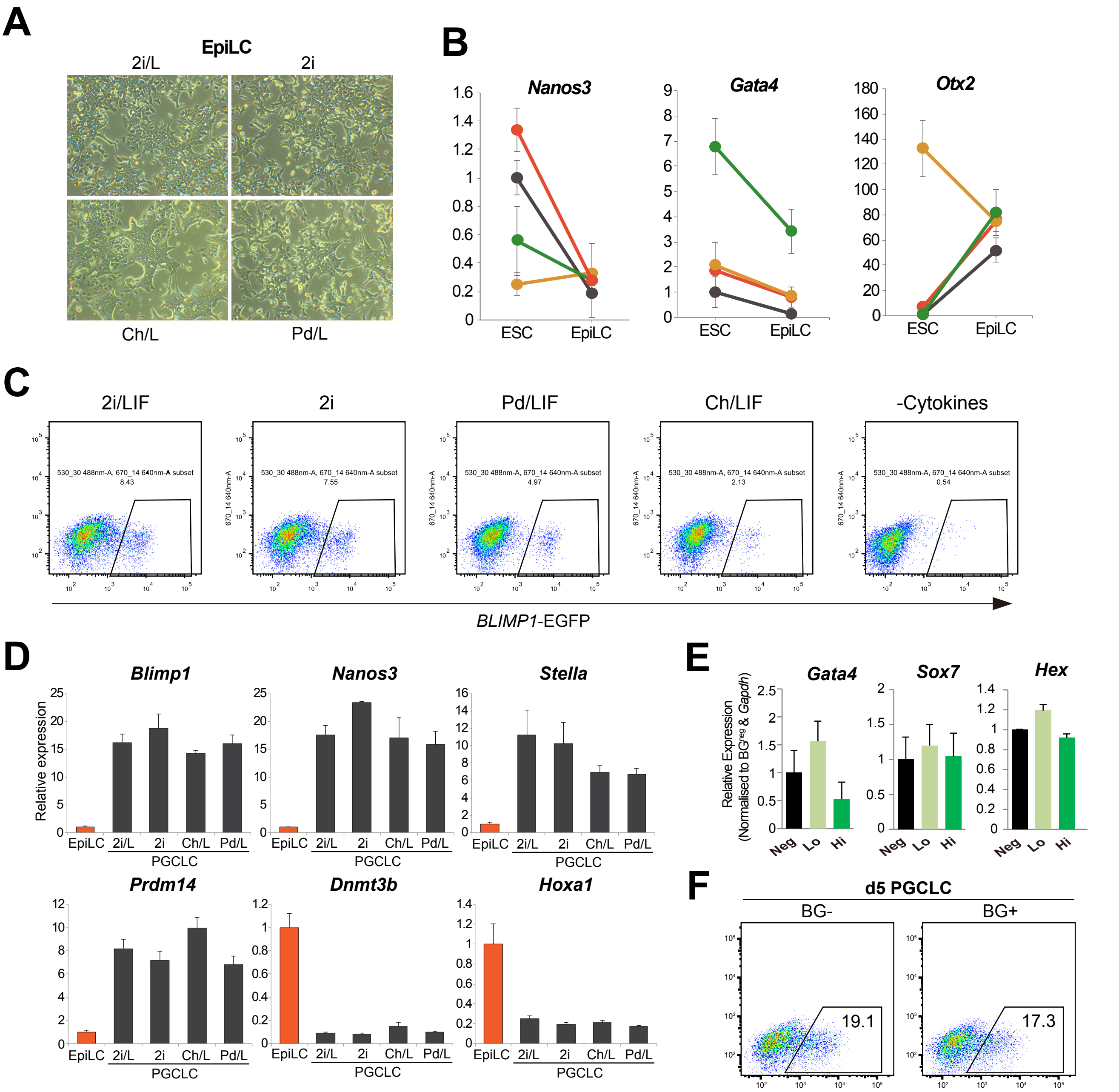

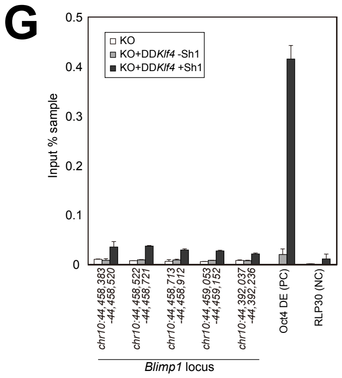

Figure S5. The initial naïve pluripotent state influences differentiation. (A) Representative images showing morphology of epiblast-like cells (EpiLC) formed from ESC cultured under the indicated condition. (B) Expression of indicated genes during EpiLC establishment. (C) Representative FACS profile of Blimp1-GFP PGCLC derived from ESC maintained in the indicated culture parameter. (D) Expression of key PGC marker genes in purified PGCLC derived from indicated naïve ESC culture suggests all initial conditions are permissive to acquire equivalent PGCLC fate but differ in their specification efficiency. (E) Expression level of primitive endoderm genes in Blimp1-GFP –negative, -low and –high ESC suggests no significant differences in these markers between the populations. (F) PGCLC generation from Blimp-GFP -negative and -positive ESC showed no difference in efficiency. (G) ChIP-qPCR showing modest/background enrichment of KLF4 at *Blimp1* cis-regulatory regions.
